# Knowledge about the health effects of cannabis before and after legalization of ‘recreational’ cannabis in Canada: findings from the International Cannabis Policy Study, 2018–2024

**DOI:** 10.1186/s42238-026-00428-6

**Published:** 2026-06-10

**Authors:** Wilson Tong, David Hammond

**Affiliations:** School of Public Health Sciences, University of Waterloo, 200 University Ave W, Waterloo, ON N2L 3G1 Canada

**Keywords:** Health knowledge, Cannabis, Canada, Legalization

## Abstract

**Background:**

Although prior research has examined how cannabis legal status relates to psychosocial predictors of use, less is known about whether recreational legalization influences public knowledge of specific cannabis-related health effects. This study addresses this gap by analyzing trends in knowledge of health effects of cannabis consumption following the legalization of recreational cannabis in Canada.

**Methods:**

Data were collected from repeated cross-sectional national surveys conducted in the year prior to legalization of ‘recreational’ cannabis in Canada (2018), and five years post-legalization (2019–2023) among 93,933 respondents aged 16–65. Regression models examined differences pre vs. post-legalization in health knowledge, assessed as agreement with each of 7 health effects from cannabis and a composite Health Knowledge Index, adjusting for cannabis consumption, mental health history, and sociodemographics.

**Results:**

Across all years, agreement was highest for the risks of impaired driving (79–81%) and lowest for psychosis and schizophrenia risk (34–38%). Health knowledge was highest among non-consumers, those without mental illness history, and youth aged 16–25 (*p* < .001, all contrasts). No changes were observed in the Health Knowledge Index pre vs post-legalization; however, significant decreases were observed for harms during pregnancy or breastfeeding (*p* < .001), danger while driving or operating machinery (*p* = 0.015), risk of psychosis and schizophrenia (*p* = 0.003), and risk of diabetes (*p* = 0.001).

**Conclusions:**

Overall, only modest differences in knowledge of cannabis health effects were observed in the 5 years following cannabis legalization in Canada; however, persistent gaps in health knowledge remained, particularly among frequent consumers.

**Supplementary Information:**

The online version contains supplementary material available at 10.1186/s42238-026-00428-6.

## Background

After more than 90 years of prohibition, legalization of recreational cannabis in Canada in 2018 marked a significant shift in federal drug policy, with broad implications for criminal justice and public health (Rosenberg et al., 2024). Cannabis legalization also has the potential to impact psychosocial predictors of cannabis consumption, including risk perceptions and health knowledge. Health knowledge encompasses the perceptions and attitudes individuals hold about the health benefits and risks of a substance, which are influenced by personal experiences, societal norms, and public health messaging (Weinstein 1999; Glanz 2015). Health knowledge is a central feature of health behaviour models and has important influence on substance use, as illustrated by the vast literature on tobacco use (Glanz 2015; Hyland et al., 2004; Curry et al., 1997; Hammond et al., 2004; US 2014). Research on tobacco control policies over the past century has demonstrated that increasing strict regulations can increase risk perceptions and decrease tobacco use (US 2014). Cannabis legalization presents a unique opportunity to examine if the reverse is also true: whether more permissive laws and policies decrease perceptions of risk.

Cannabis health knowledge and risk perceptions are inversely associated with consumption and future susceptibility to consume cannabis (Salloum et al., 2018; Pacek et al., 2015). The association between risk perception and consumption is bi-directional: lower perceptions of risk increase the likelihood of future consumption, whereas frequent consumption can undermine risk perceptions as a result of defensive avoidance and optimism bias (Mader et al., 2022; McMahon et al., 2023; Salloum et al., 2018; Leos-Toro 2019). To date, few studies have assessed the impact of cannabis legalization on risk perceptions and health knowledge. Nationally representative monitoring surveys observed increased health knowledge between 2018 and 2023 for beliefs about cannabis addictiveness, with decreases observed for beliefs about the risks of cannabis consumption during pregnancy, teenage consumption, harmfulness of cannabis, and association with mental health problems (Government 2024). Surveys conducted among Canadian youth found no immediate changes in risk perceptions among young adults two months after legalization (Robinson et al., 2020).

In the United States, several studies have examined risk perceptions following state-level changes in cannabis laws, with mixed findings. National surveys conducted with high school students indicate a decline in risk perceptions for cannabis between 1991 and 2006, which includes the period in which several states legalized medical cannabis consumption and the earliest states to adapt recreational legalization (Sarvet et al., 2018). However, most pre-post studies in states that have legalized cannabis show little or no effect. For example, no differences in risk perceptions were observed among youth and young adults before and after medical cannabis legalization in Massachusetts, similar to null findings before and after recreational cannabis legalization in Colorado, and in difference-in-differences models examining cannabis laws across 22 states (Mennis et al., 2023; O’Connell et al., 2022; Harpin et al., 2018). Mixed findings have also been observed in studies that examined changes in risk perceptions before and after ‘recreational’ legalization in Washington State (Harpin et al., 2018; Estoup et al., 2016; Bailey et al., 2020; Blevins et al., 2018). Interpreting state-level differences is complicated by pre-existing differences between states that did and did not implemented cannabis legalization (Blevins et al., 2018; Wall et al., 2011). As such, the impact of legalization on risk perceptions of cannabis consumption remains unclear (Goodman and Hammond 2022).

Reductions in perceived risks—and potential increases in cannabis consumption among youth—was among the public health concerns identified in the lead up to legalization in Canada (Task 2016). In addition to speculation that legalization would undermine risk perceptions, there were specific concerns about the impact of commercializing the cannabis market and the impact of industry marketing. Decades of research in tobacco control demonstrated that the positive imagery conveyed in industry marketing was highly effective in increasing social norms and decreasing perceptions of risk, both of which are key mediators in the causal effect of tobacco marketing on youth smoking (National 2012). Conversely, legalization provides new opportunities for conveying information on the risks of cannabis consumption. Most notably, licensed cannabis products in Canada are mandated to display health warning labels on packages. Packaging must display a series of ‘rotating’ warnings which convey different messages, including the effects of cannabis on mental health, among young people, and impaired driving (Health 2025). Literature reviews of health warnings on tobacco products has demonstrated that comprehensive warnings are effective in increasing knowledge of specific health effects and risk perceptions more generally (Goodman et al., 2021; Massey et al., 2024). Emerging research on health warnings for cannabis is generally consistent with the literature on tobacco products, but at a relatively early stage, with few population-level studies to date (Leos-Toro 2019; Government 2024; Massey et al., 2024; Goodman et al., 2022). In Canada, recall of cannabis health warnings has increased since legalization, particularly among frequent consumers (Goodman et al., 2021), however, it is unclear how these effects may translate into population-level indicators of health knowledge. Overall, while there are strong conceptual grounds to suggest that specific regulatory measures may impact perceptions of risk as part of a broader legal framework, this area is largely unexplored.

Overall, cannabis legalization has potential to influence a key predictor of cannabis consumption among youth and adults; however, research to date includes few pre-post research designs and is predominantly focussed on risk perceptions among young people, with a lack of studies among the general adult population This represents an important evidence gap given that cannabis consumption in older adults has been increasing and potentially different risk profile compared to younger cohorts (Han et al., 2021; Health 2023; Huỳnh et al., 2024; Hendrickson et al., 2020; Kuerbis 2019). In addition, most studies have assessed general perception of risk or harm, rather than specific health beliefs. This is notable because many of the risk communications in Canada and other legal markets convey specific health effects as part of mandated warning labels on products and through mass media campaigns targeting impaired driving or use among young people.

The current study sought to examine changes in population-level knowledge of the health effects of cannabis before and in the 5-years following legalization in Canada. The study also examined potential differences in the knowledge of health risks of cannabis consumption by frequency of cannabis consumption, mental health status, age, educational attainment, and income adequacy.

## Methods

Data are from Waves 1–6 of the Canadian component of the International Cannabis Policy Study (ICPS). National online surveys were conducted annually between 2018 and 2023 with respondents aged 16–65. Respondents were sampled through the Nielsen Consumer Insights Global Panel and partner panels. The Nielsen panels are recruited using a variety of probability and non-probability sampling methods. For the ICPS surveys, Nielsen draws stratified random samples from online panels, with quotas based on age and state/province of residence. Upon completion, respondents receive remuneration in accordance with their panel’s usual incentive structure. Monetary incentives have been shown to increase response rates and decrease response bias in subgroups under-represented in surveys, including disadvantaged subgroups. The cooperation rate, which was calculated based on AAPOR Cooperation Rate #2 as the percentage of respondents who completed the survey of eligible respondents those who accessed the survey link, ranged between 53.8% to 64.2% across the six survey waves. Surveys were conducted in English or French, with a median survey time ranging between 20 to 23 min across years.

Respondents provided consent prior to completing the survey. Respondents received remuneration in accordance with their panel’s usual incentive structure (e.g., points based or monetary rewards, chances to win prizes). A full description of the study methods can be found in the ICPS Technical Reports www.cannnabisproject.ca/methods and methodology paper (Hammond et al., 2020).

### Measures

All survey measures are publicly accessible on the ICPS website https://cannabisproject.ca/publications/

#### Health knowledge

Knowledge of the health effects of cannabis was assessed using measures of agreement with a list of seven health effects, with the response options “yes”, “no”, “maybe”. Six health effects were selected based on systematic reviews of health effects and those featuring in mandated health warnings under the Cannabis Act (Hammond et al., 2020; Abrams 2018). In addition, one “false” health belief question that was included for which there is no clear evidence: “Can using marijuana cause diabetes?”. Verbatim wording for the health knowledge questions is included in Supplemental Table 6.

An index variable was created by summing the number of correct responses across the seven items included in all years, called the Health Knowledge Index (range 0–14). Responses that were “true” health statements were coded as “Correct” if respondents selected “Yes”, while responses to “false” health statements were coded as “Correct” if respondents selected “No”. Correct responses had a value of 2, while “Maybe/Don’t Know” and incorrect responses had values of 1 and 0, respectively.

#### Sociodemographics

Respondents provided sociodemographic information, including sex at birth, age, ethnicity/race, highest education level, and perceived income adequacy. Age was classified into five categories: 16–25, 26–35, 36–45, 46–55, and 56–65. Ethnicity/race was classified with Canadian-specific measures drawn from the census or benchmark health surveys. Income adequacy was assessed by asking respondents, “Thinking about your family’s income, how difficult or easy is it to make ends meet?”, to which responses ranged on a 5-point Likert scale from very difficult to very easy (Acton et al., 2025).

#### Cannabis consumption

Respondents were classified into six categories based on the status of cannabis consumption: Never consumed, consumed more than 1 month ago, monthly/weekly consumption, daily/almost daily consumption (≥ 5 days per week).

#### Mental health status

Mental health status was assessed by measuring whether respondents had been diagnosed with any of the mental health conditions in a provided list (see Supplemental Table 7). Responses were classified into 3 categories (No; Yes; Not stated – including Refuse to Answer and Don’t Know responses).

#### Noticing of cannabis product health warnings, advertisements, and education campaigns

Population-level health communication campaigns have the potential to influence health knowledge, three of which were assessed in the current study. First, noticing of cannabis product health warnings was assessed using the question “In the past 12 months, have you seen health warnings on marijuana products or packages?”, with responses classified as (Yes, No, Not applicable – I have not seen any marijuana products or packages, Don’t know, Refuse to answer) (Goodman et al., 2022). Noticing cannabis advertising from any of sixteen channels was assessed using a derived version of the question “In the past 12 months, have you noticed marijuana being advertised or promoted in any of the following places?”, with responses classified as (Yes, No, Don’t know, Refuse to answer) (Winfield-Ward et al., 2025). Noticing cannabis education campaigns from any of eighteen channels was assessed using a derived version the question “In the past 12 months, have you noticed education campaigns or public health messages about marijuana in any of the following places?”, with responses classified as (Yes, No, Don’t know, Refuse to answer). All measures are shown in Supplemental Table 8.

### Analysis

A total of 93,933 respondents completed ICPS surveys between 2018 to 2023. The current analysis consisted of a sub-sample of 92,365 after excluding 1,568 respondents who refused to answer any questions on noticing cannabis product health warnings, advertisements, educational campaigns or any health knowledge questions.

All analyses present weighted data. Post-stratification sample weights based on sex, age, region, education, race and consumption status were constructed based on the Canadian Census estimates and a raking algorithm applied to the original samples; see the Technical Reports for details (http://cannabisproject.ca/methods/). Weights were rescaled to the unweighted analytic sample size within each wave.

Separate logistic regression models were run for each of the seven health belief questions. Models were run in two steps. First, “unadjusted” models included only the indicator variable for survey year to examine trends over time. Contrasts between 2018 (the ‘baseline’ prior to legalization) and each of the subsequent years are reported in the Results section to highlight ‘pre – post’ changes. Pairwise contrasts between all years are also reported in supplemental tables. Second, “adjusted” models were run, which included the following covariates: survey year, cannabis consumption status, mental health diagnosis, age, sex, education, ethnicity, income adequacy. Sensitivity analyses run for each of the seven health beliefs, in which each health belief question was analyzed as a 3-level variable (“Maybe”/”Don’t know” was analyzed as a separate category from “No” responses) showed the same general pattern of significance as the main models. Odds ratios (AORs) and 95% confidence intervals (CIs) are shown. Analyses were conducted using survey procedures in SAS 8.4.

A linear regression model was run to examine changes in the Health Knowledge Index (range = 0–14; higher scores indicate more correct responses). Unadjusted and adjusted models were run, as described above. In an additional step, the three policy-related variables were added to the adjusted models: noticing cannabis health warnings (Yes; No; Don’t Know; Not applicable; Refuse to Answer), noticing cannabis advertisements (Noticed no ads; Noticed an ad in at least 1 channel; Don’t Know), and noticing of public health campaigns (Noticed no education campaigns/public health messages, Noticed an education campaign/public health message in at least 1 channel; Don’t Know).

## Results

### Sample characteristics

As Table 1 shows, the sample included approximately equal proportions in each age group and females/males. A majority of respondents were White and held a bachelor's degree or higher, and most respondents had consumed cannabis at least once in their lifetime.

**Table 1:** Weighted sample characteristics (*n* = 92,365)

**Characteristic**	**2018** (*n* = 9954)	**2019** (*n* = 15,007)	**2020** (*n* = 15,531)	**2021** (*n* = 16,683)	**2022** (*n* = 15,650)	**2023** (*n* = 19,540)	**Total** (*n* = 92,365)
Age							
16–25 years	18.8% (1875)	18.5% (2782)	18.5% (2873)	18.2% (3028)	18.5% (2900)	18% (3516)	18.4% (16,974)
26–35 years	20.6% (2052)	20.6% (3093)	20.8% (3232)	21.0% (3501)	21.0% (3289)	21.3% (4156)	20.9% (19,323)
36–45 years	19.4% (1934)	19.8% (2978)	20.0% (3099)	20.3% (3382)	20.4% (3199)	20.6% (4024)	20.2% (18,615)
46–55 years	21.1% (2176)	20.1% (3010)	19.6% (3043)	19.3% (3224)	19.0% (2968)	19.1% (3735)	19.6% (18,069)
56–65 years	20.1% (2090)	21.0% (3145)	21.1% (3285)	21.3% (3548)	21.0% (3293)	21.0% (4109)	21% (19,384)
Sex at Birth							
Female	50.0% (4977)	49.9% (7487)	50.1% (7775)	49.9% (8326)	49.9% (7808)	50.0% (9776)	50.0% (46,149)
Male	50.0% (4977)	50.1% (7520)	49.9% (7756)	50.1% (8357)	50.1% (7842)	50.0% (9764)	50.0% (46,216)
Ethnicity							
White only	77.5% (7716)	73.6% (11,046)	72% (11,109)	68.4% (11,415)	67.5% (10,567)	66.9% (13,064)	70.3% (64,916)
East/South East Asian only	8.5% (842)	7.7% (1163)	9.1% (1409)	8.5% (1426)	8.8% (1383)	9.1% (1782)	8.7% (8004)
Mixed/Other/Unstated	3.8% (382)	6.8% (1018)	6.8% (1052)	7.3% (1212)	7.5% (1177)	7.4% (1451)	6.8% (6291)
South Asian only	3.1% (305)	3.2% (479)	3.7% (580)	4.4% (730)	4.6% (719)	5.5% (1070)	4.2% (3882)
Black only	1.6% (156)	3.6% (545)	3.5% (543)	4.0% (668)	5.0% (775)	5.0% (979)	4.0% (3665)
Indigenous only	3.7% (367)	2.3% (347)	1.8% (285)	2.6% (441)	2.4% (371)	2.4% (468)	2.5% (2278)
Latino only	0.9% (88)	1.5% (219)	1.8% (278)	2.8% (468)	2.1% (328)	1.6% (321)	1.8% (1700)
Middle Eastern only	1.0% (99)	1.3% (192)	1.8% (276)	1.9% (324)	2.1% (331)	2.1% (406)	1.8% (1627)
Income Adequacy							
Very difficult	8.2% (815)	9.6% (1434)	7.5% (1167)	8.7% (1459)	10.2% (1604)	12.1% (2357)	9.6% (8834)
Difficult	20.1% (2001)	22.4% (3354)	18.5% (2869)	19.0% (3175)	22.1% (3459)	23.6% (4614)	21.1% (19,473)
Neither easy nor difficult	36.0% (3583)	35.1% (5260)	37.5% (5821)	35.1% (5858)	34.1% (5341)	33.8% (6599)	35.1% (32,461)
Easy	21.2% (2112)	19.8% (2967)	22.1% (3437)	21.5% (3584)	20.1% (3146)	18.8% (3671)	20.5% (18,918)
Very easy	11.2% (1118)	9.6% (1444)	10.8% (1674)	12.1% (2020)	10.1% (1585)	9.1% (1776)	10.4% (9617)
Not stated*	3.3% (325)	3.6% (547)	3.6% (562)	3.5% (588)	3.3% (516)	2.7% (524)	3.3% (3062)
Education							
Less than high school	15.3% (1524)	15.4% (2309)	15.2% (2358)	15.2% (2541)	13.3% (2076)	13.5% (2609)	14.5% (13,417)
High school diploma or equivalent	26.7% (2656)	26.5% (3976)	26.5% (4112)	26.4% (4405)	26.1% (4079)	25.9% (5062)	26.3% (24,290)
Bachelor's degree or higher	57.4% (5712)	57.2% (8586)	57.3% (8895)	57.4% (9568)	59.6% (9324)	59.7% (11,665)	58.2% (53,749)
Not stated	0.6% (62)	0.9% (136)	1.1% (166)	1.0% (170)	1.1% (170)	1.0% (205)	1.0% (909)
Mental Health Diagnosis							
No	72.6% (7223)	61.0% (9155)	60.3% (9362)	57.6% (9605)	56.4% (8832)	57.7% (11,280)	60.0% (55,457)
Yes	26.2% (2603)	34.9% (5236)	34.3% (5331)	36.6% (6113)	37.9% (5924)	37.4% (7317)	35.2% (32,525)
Not stated*	1.3% (128)	4.1% (615)	5.4% (838)	5.8% (965)	5.7% (895)	4.8% (942)	4.7% (4383)
Cannabis Consumption							
Never consumed	43.7% (4345)	38.3% (5741)	39.5% (6133)	38.0% (6343)	38.5% (6027)	38.3% (7476)	39.0% (36,066)
Consumed more than 1 month ago	37.8% (3758)	38.3% (5748)	37.1% (5757)	35.3% (5885)	36.6% (5732)	36.5% (7128)	36.8% (34,008)
Monthly/Weekly consumption	9.8% (972)	12.4% (1862)	11.9% (1841)	13.2% (2195)	13.0% (2031)	13.0% (2545)	12.4% (11,446)
Daily/almost daily consumption	8.8% (879)	11.0% (1657)	11.6% (1800)	13.5% (2259)	11.9% (1859)	12.2% (2391)	11.7% (10,845)

^*^Includes “Refuse to answer” & “Don’t Know” responses

### Knowledge of individual health effects

**Fig. 1:**
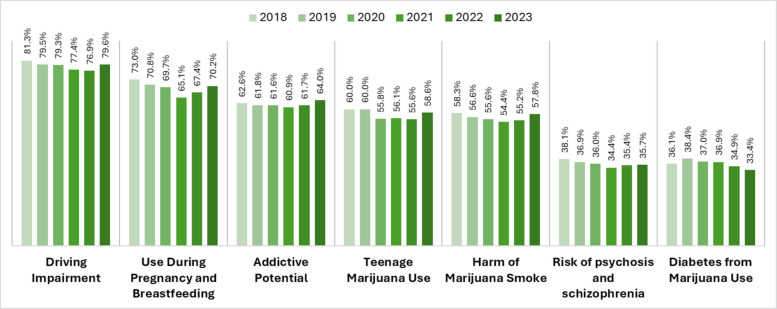
Frequency of correct responses to cannabis health knowledge questions over time (*n* = 92,365)

**Fig. 2:**
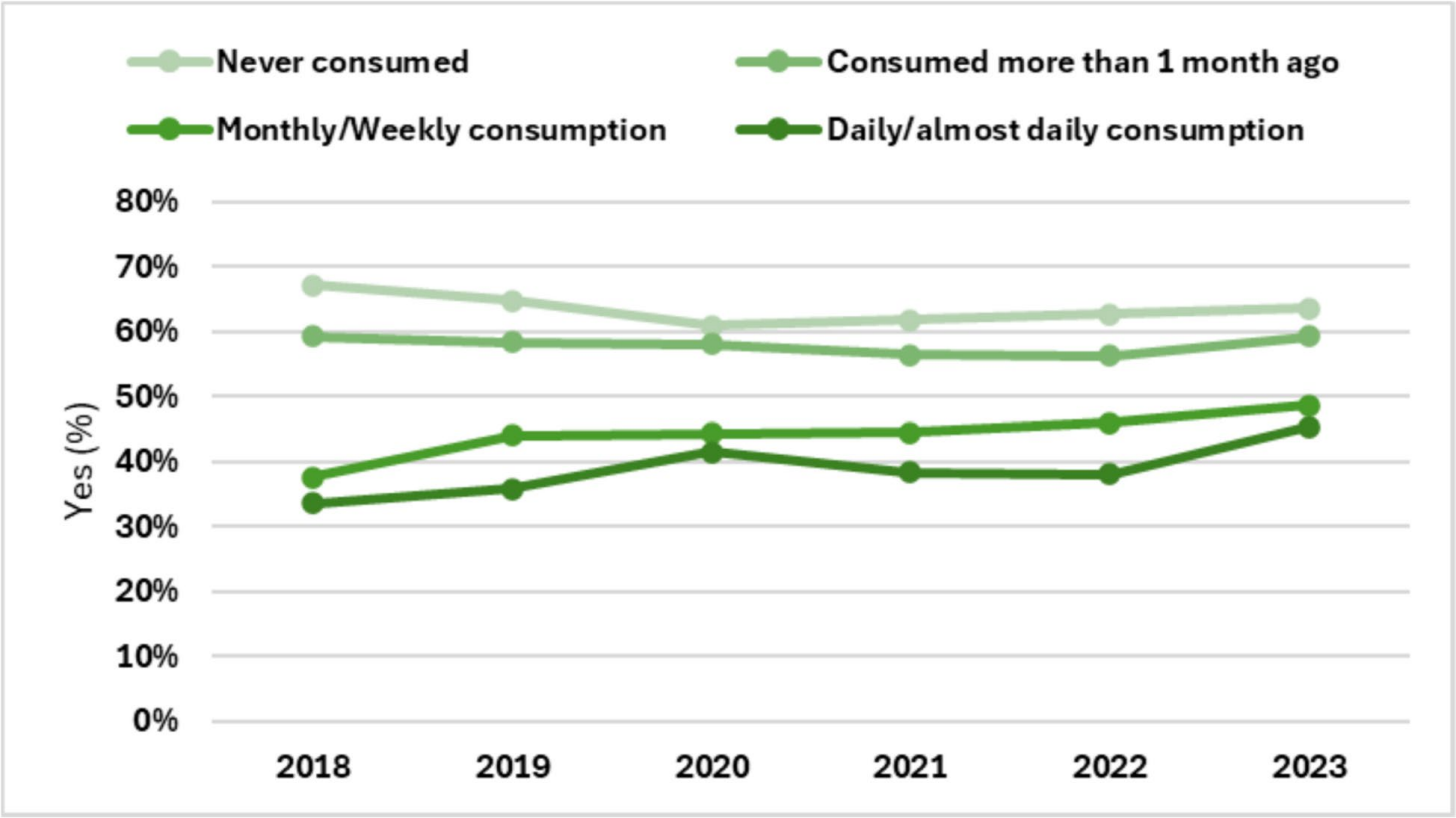
Belief that marijuana smoke can be harmful, by cannabis consumption status (*n* = 92,365)

**Fig. 3:**
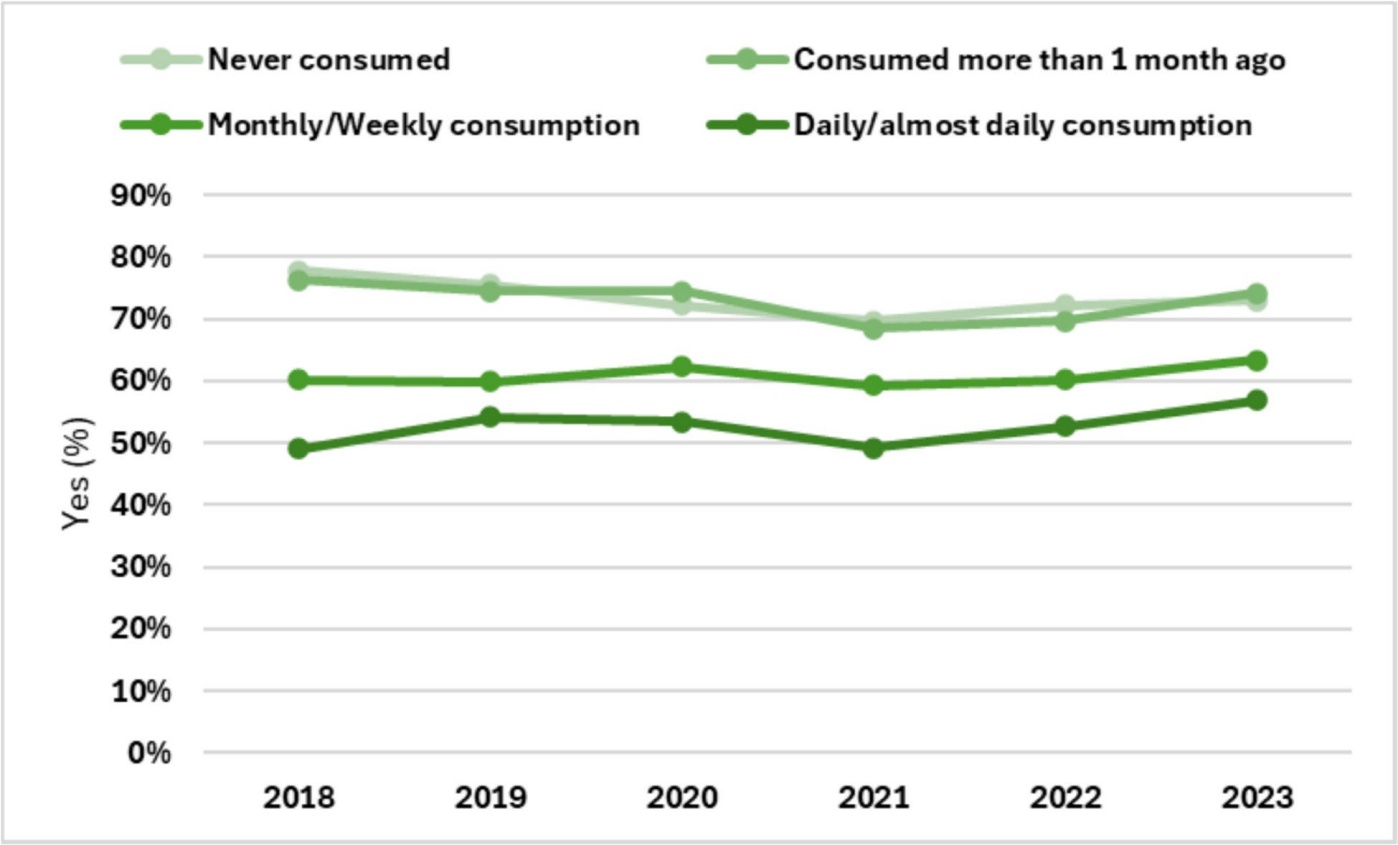
Belief that marijuana consumption can be harmful while pregnant or breastfeeding, by cannabis consumption status (*n* = 92,365)

**Fig. 4:**
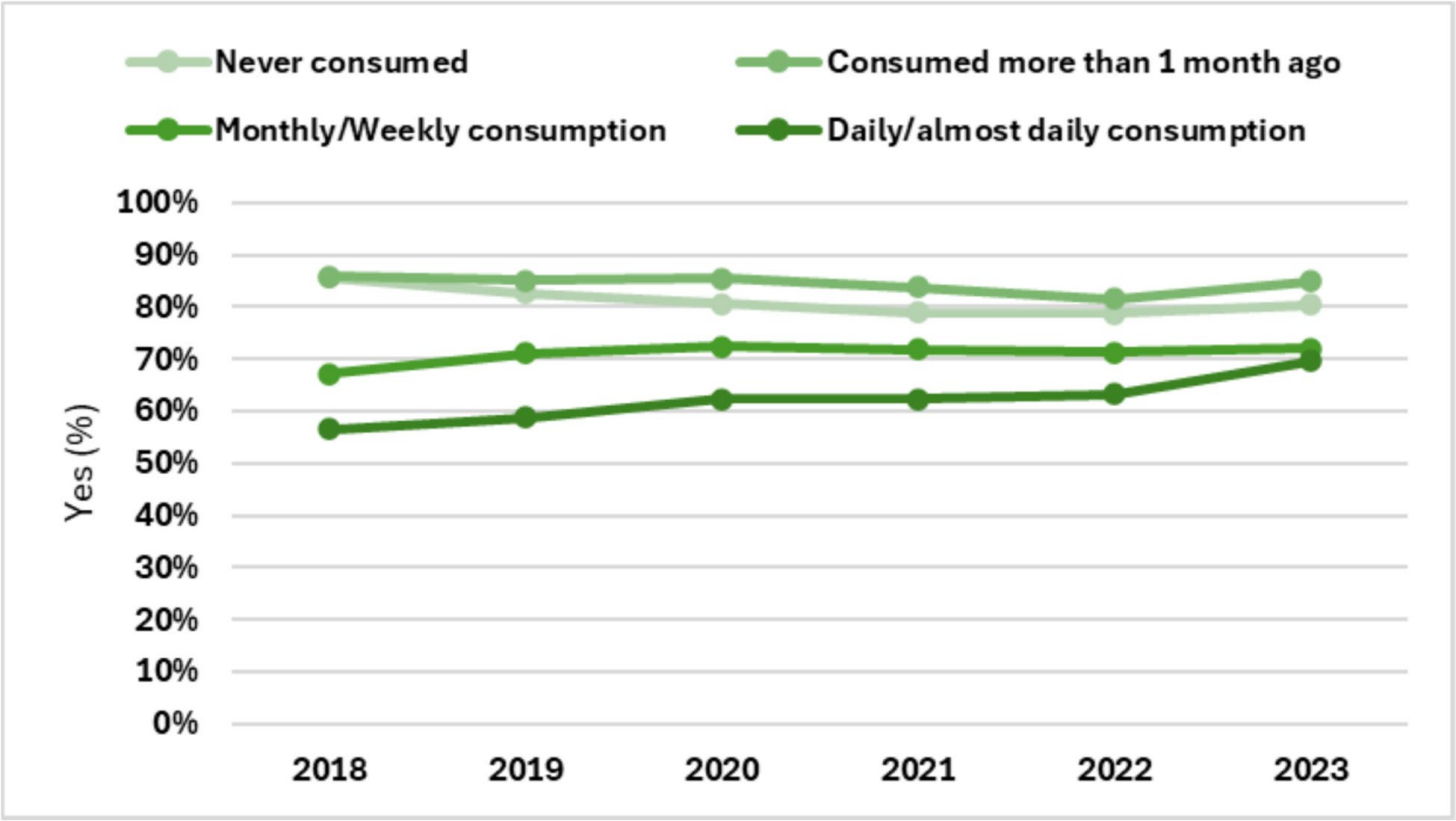
Belief that it can be dangerous to drive or operate machinery after using marijuana, by cannabis consumption status (*n* = 92,365)

**Fig. 5:**
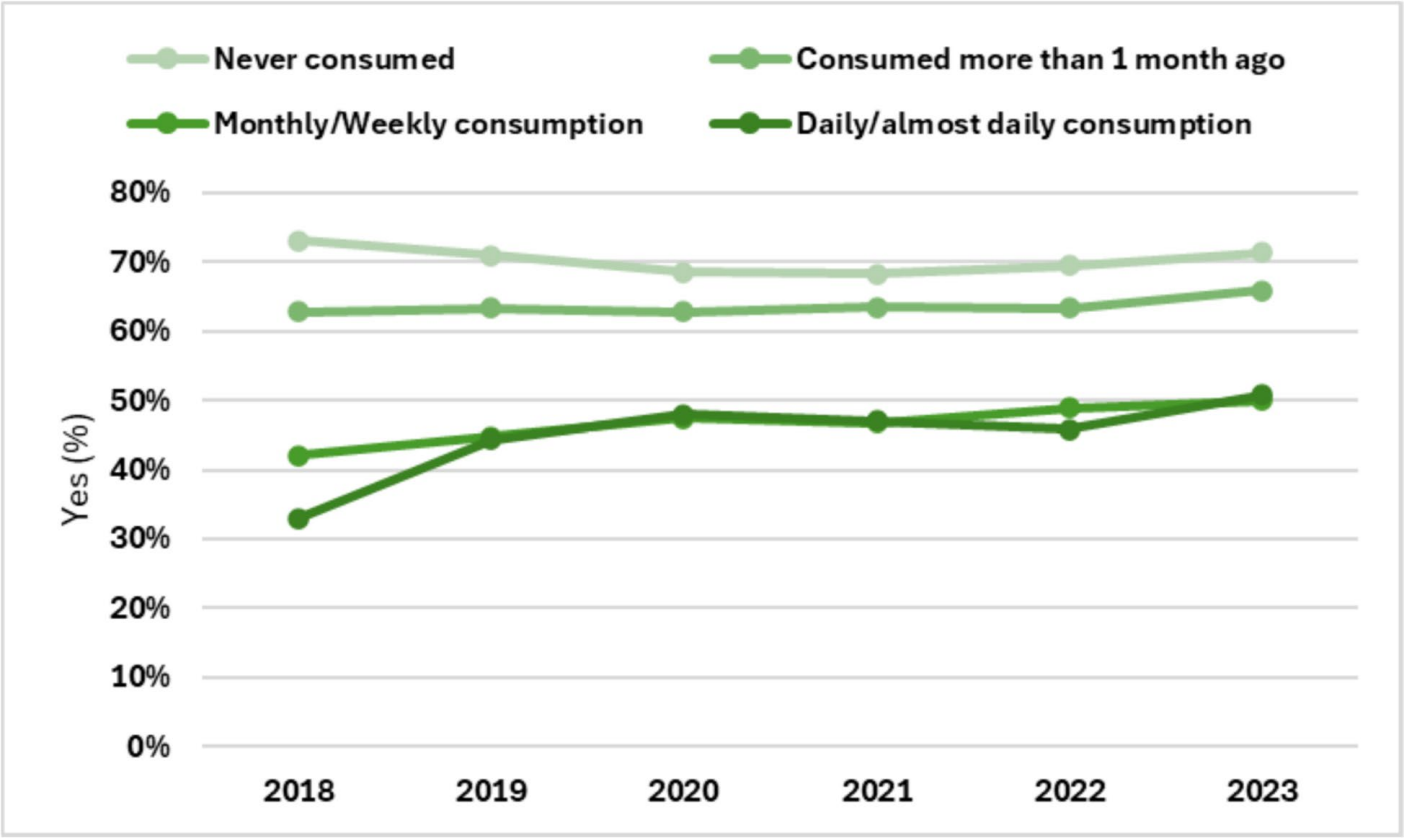
Belief that marijuana can be addictive, by cannabis consumption status (*n* = 92,365)

**Fig. 6:**
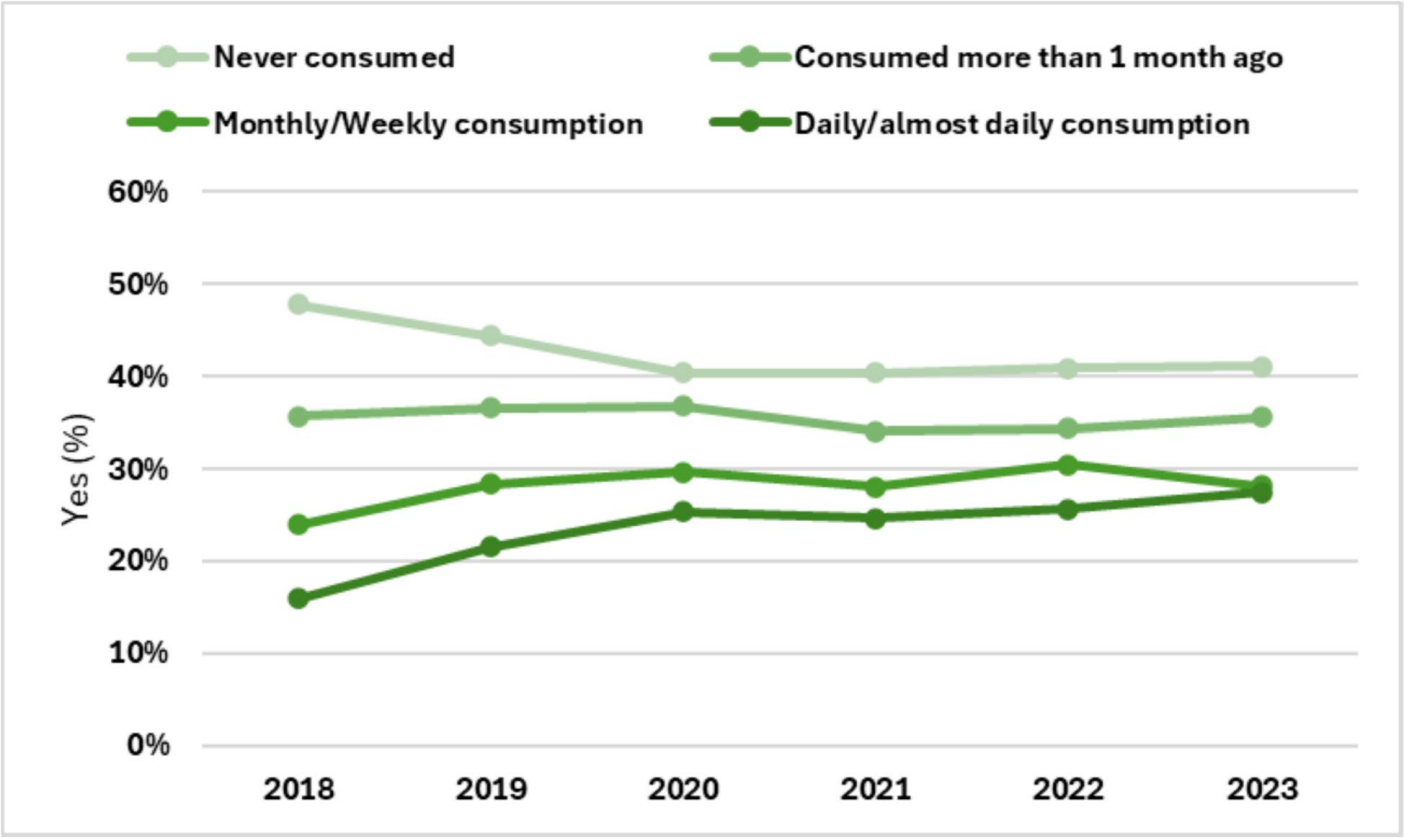
Belief that marijuana use can increase the risk of psychosis and schizophrenia, by cannabis consumption status (*n* = 92,365)

**Fig. 7:**
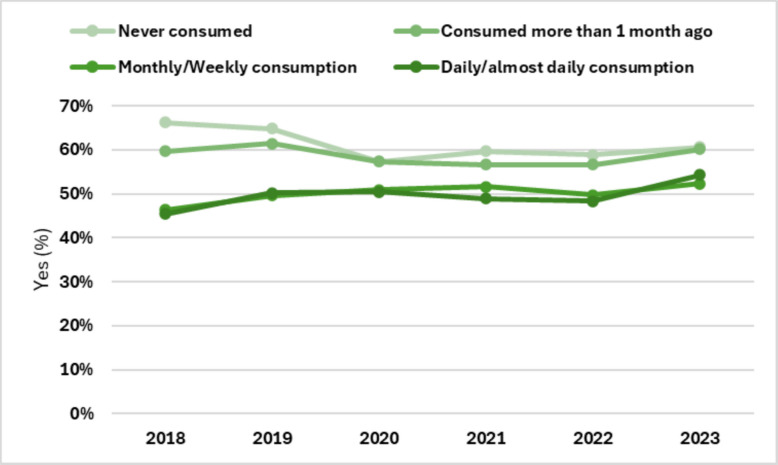
Belief that teenagers are at greater risk of harm from marijuana use than adults, by cannabis consumption status (*n* = 92,365)

**Fig. 8:**
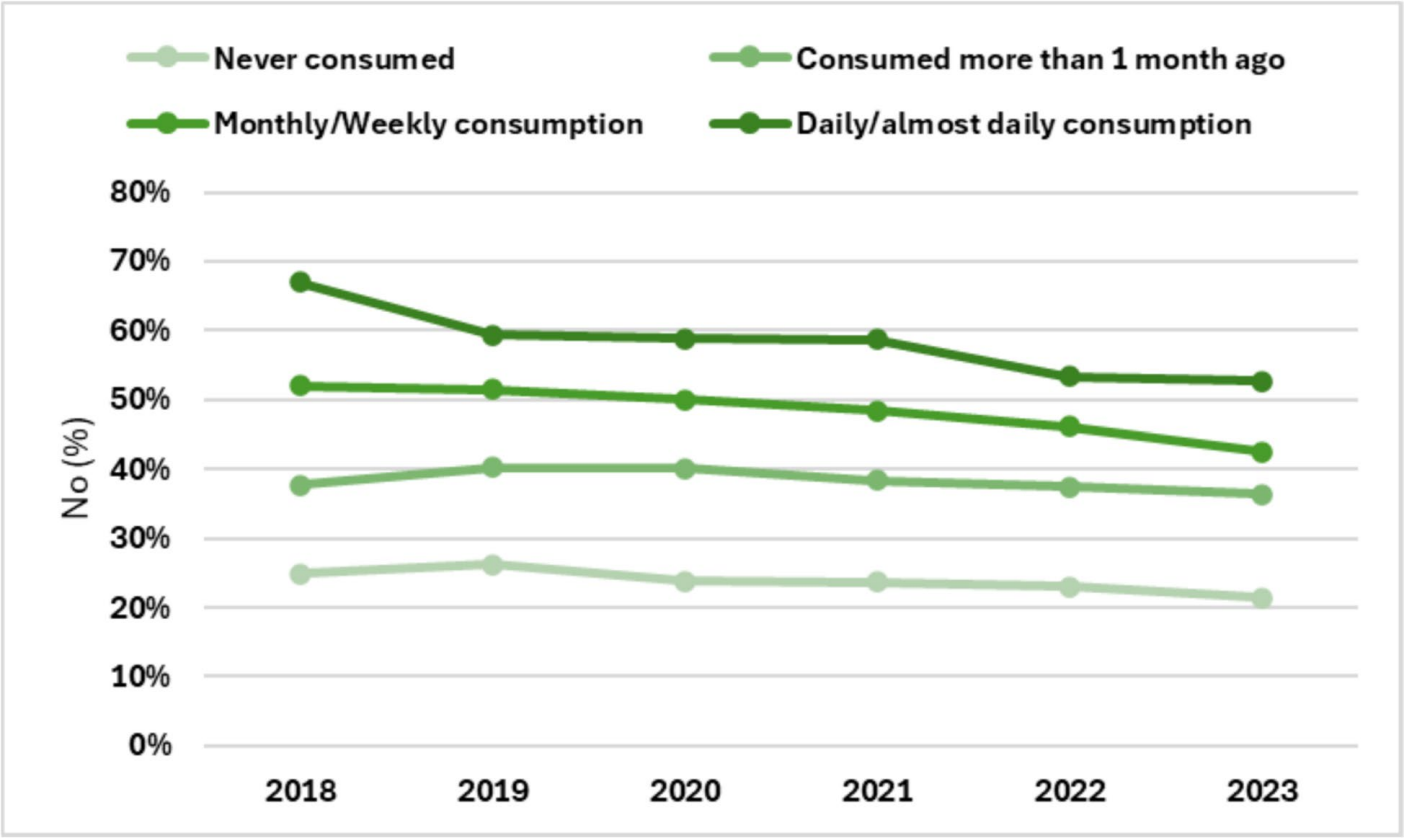
Belief that marijuana use can cause diabetes, by cannabis consumption status (*n* = 92,365)

Figure 1 shows levels of agreement for each of the 7 health effects, by year. Differences in health knowledge observed by cannabis consumption status are shown in Figs. 2, 3, 4, 5, 6, 7, 8. Across all years, health knowledge was highest for beliefs about driving impairment and consumption during pregnancy and breastfeeding, and lowest for beliefs about risk of psychosis and schizophrenia and diabetes from marijuana use. (See Supplemental Table 2 for responses by survey year and frequency of cannabis consumption.).

Supplemental Tables 3.1 to 3.8 show unadjusted and adjusted models testing changes over time for each health belief. Knowledge levels were lower in 2023 compared to 2018 for the effects of marijuana consumption during pregnancy and breastfeeding (2018 = 72.9% vs. 2023 = 70.2%; OR = 0.87, 0.81–0.94; *p* < 0.001), driving impairment (2018 = 81.3% vs. 2023 = 79.6%; OR = 0.90, 0.82–0.98; *p* = 0.015), risk of psychosis and schizophrenia (2018 = 38.1% vs. 2023 = 35.7%; OR = 0.90, 0.84–0.97; *p* = 0.003), and diabetes (2018 = 36.1% vs. 2023 = 33.4%; OR = 1.25, 1.18–1.32; *p* < 0.001). No differences were observed for beliefs on the harm of marijuana smoke (2018 = 58.3% vs. 2023 = 57.8%; OR = 0.98, 0.92–1.05; *p* = 0.570), addictive potential (2018 = 62.6% vs 2023 = 64.0%; OR = 0.98, 0.99–1.14; *p* = 0.080), teenage marijuana consumption (2018 = 60.0% vs 2023 = 58.6%; OR = 0.94, 0.88–1.01; *p* = 0.087) in unadjusted models. However, in the adjusted models knowledge levels were lower in 2023 compared to 2018 for only and risk of diabetes (AOR = 0.85, 0.79–0.91; *p* < 0.001). Moreover, knowledge levels were higher in 2023 compared to 2018 for addictive potential (OR = 1.18, 1.10–1.23; *p* < 0.001), while no differences were observed for other beliefs.

### Health knowledge index

In the unadjusted linear regression model, compared to Health Knowledge Index sores in 2018, scores were significantly lower in 2020 (β = −0.12, −0.21- −0.03; *p* = 0.006), 2021 (β = −0.25, −0.33- −0.17; *p* < 0.001), and 2022 (β = −0.23, −0.32- −0.15; *p* < 0.001), with no significant differences observed compared to other years. Pairwise contrasts for all other years are shown in Supplemental Table 4.

Findings from the adjusted linear regression models were somewhat different than the unadjusted models. As shown in Table 2, compared to 2018, scores on the Health Knowledge Index were significantly higher in 2019 (β = 0.09, 0.01—0.17; *p* = 0.032) and 2023 (β = 0.14;0.06—0.22, *p* < 0.001). Pairwise contrasts for all other years are shown in Supplemental Table 5.

**Table 2:** Adjusted linear regression model examining the Health Knowledge Index (*n* = 92,365)

Characteristic	Mean (SD)*	Beta (95% CI)	*P*-value
Survey year			
2018	10.6 (2.4)	ref	ref
2019	10.5 (2.5)	0.09 (0.01, 0.17)	0.032
2020	10.5 (2.5)	0.03 (−0.05, 0.11)	0.478
2021	10.3 (2.6)	−0.04 (−0.12, 0.04)	0.319
2022	10.4 (2.5)	−0.05 (−0.13, 0.03)	0.255
2023	10.5 (2.4)	0.14 (0.06, 0.22)	<.001
Cannabis Consumption			
Never consumed	10.8 (2.4)	ref	ref
Consumed more than 1 month ago	10.7 (2.2)	−0.21 (−0.25, −0.16)	<.001
Monthly/Weekly consumption	9.7 (2.8)	−1.00 (−1.07, −0.92)	<.001
Daily/almost daily consumption	9.4 (2.9)	−1.30 (−1.38, −1.22)	<.001
Mental Health Diagnosis			
No	10.7 (2.3)	ref	ref
Not stated	9.1 (3.0)	−1.13 (−1.25, −1.02)	<.001
Yes	10.3 (2.6)	−0.15 (−0.20, −0.11)	<.001
Sex			
Female	10.7 (2.1)	ref	ref
Male	10.2 (3.0)	−0.40 (−0.44, −0.36)	<.001
Age			
16–25 years	10.5 (2.9)	ref	ref
26–35 years	10.0 (2.9)	−0.59 (−0.67, −0.51)	<.001
36–45 years	10.3 (2.6)	−0.39 (−0.47, −0.32)	<.001
46–55 years	10.7 (2.3)	−0.12 (−0.19, −0.04)	0.002
56–65 years	10.8 (1.9)	−0.16 (−0.23, −0.09)	<.001
Ethnicity			
White only	10.6 (2.4)	ref	ref
East/South East Asian only	10.4 (2.5)	−0.39 (−0.46, −0.32)	<.001
Mixed/Other/Unstated	10.0 (2.8)	−0.35 (−0.44, −0.26)	<.001
South Asian only	10.2 (2.8)	−0.57 (−0.67, −0.46)	<.001
Black only	9.9 (2.9)	−0.59 (−0.70, −0.47)	<.001
Indigenous only	9.7 (2.9)	−0.55 (−0.70, −0.40)	<.001
Latino only	10.0 (2.9)	−0.53 (−0.70, −0.36)	<.001
Middle Eastern only	10.2 (2.9)	−0.47 (−0.64, −0.30)	<.001
Income Adequacy			
Very difficult	10.3 (2.6)	ref	ref
Difficult	10.5 (2.5)	0.09 (0.01, 0.17)	0.036
Neither easy nor difficult	10.4 (2.5)	−0.05 (−0.13, 0.03)	0.235
Easy	10.7 (2.4)	0.13 (0.05, 0.22)	0.002
Very easy	10.7 (2.5)	0.10 (0.01, 0.20)	0.033
Not stated	9.5 (2.9)	−0.62 (−0.76, −0.48)	<.001
Education			
Less than high school	10.3 (3.5)	ref	ref
High school or equivalent	10.2 (3.3)	0.01 (−0.08, 0.09)	0.890
Bachelor's degree or higher	10.6 (2.1)	0.42 (0.34, 0.49)	<.001
Not stated	8.9 (3.0)	−0.57 (−0.81, −0.33)	<.001

^*^Unadjusted means and standard deviations are reported in this table

Compared to respondents who reported having no mental health diagnoses, Health Knowledge Index scores were significantly lower among those who reported 1 or more mental health diagnoses (β = −0.15, −0.20—−0.11, *p* < 0.001) and those who did not respond to this question (β = −1.13, −1.25—−1.02, *p* < 0.001). Compared to respondents who reported 1 or more mental health diagnoses scores were lower among those who did not report (β = −0.98, −1.10—−0.86, < 0.001).

Younger age was associated with higher Health Knowledge Index scores. Compared to 16–25-year-old respondents, scores were significantly lower among all other age groups, including 26–35-year-old (β = −0.59, −0.67—−0.51, *p* < 0.001), 36–45 year old (β = −0.39, −0.47—−0.32, *p* < 0.001), 46–55 year old (β = −0.12, −0.19—−0.04, *p* = 0.002), and 56–65 year old respondents (β = −0.16, −0.23—−0.09, *p* < 0.001). Females also reported greater health knowledge than males (β = 0.40, 0.36–0.44, *p* < 0.001). Ethnicity was also significant associated with health knowledge. Compared to respondents who identified as White only, scores were lower for all other identified ethnicities including East/South East Asian only (β = −0.39, −0.46 – −0.32, *p* < 0.001), South Asian only (β = −0.57, −0.67 – −0.46, *p* < 0.001), Black only (β = −0.59, −0.70 – −0.47, *p* < 0.001), Indigenous only (β = −0.55, −0.70 – −0.40, *p* < 0.001), Latino only (β = −0.53, −0.70 – −0.36, *p* < 0.001), Middle Eastern only (β = −0.47, −0.64 – −0.30, *p* < 0.001), and those who identified as mixed, other, or did not state (β = −0.35, −0.44 – −0.26, *p* < 0.001).

Health knowledge was generally positively associated with income adequacy and education level. For example, respondents who reported “very difficult” income adequacy reported lower Health Knowledge Index scores than those who reported their income adequacy as difficult (β = 0.09, 0.01—0.17; *p* = 0.036), easy (β = 0.13, 0.05—0.22; *p* = 0.002) and very easy (β = 0.10, 0.01—0.20; *p* = 0.033). Scores were also lower among respondents with less than a high school diploma compared to those with a bachelor's degree or higher (β = 0.42, 0.34–0.49; *p* < 0.001). In addition, scores among those who did not state their education were significantly lower compared to all other groups, including those with less than a high school diploma (β = −0.57, −0.81—−0.33; *p* < 0.001), those with a high school diploma or equivalent (β = −0.58, −0.81—−0.34; *p* < 0.001), and those with a bachelor’s degree or higher (β = −0.99, −1.21—−0.76; *p* < 0.001).

**Fig. 9:**
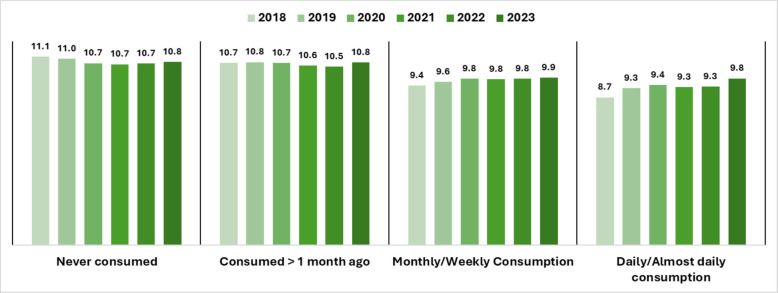
Mean Health Knowledge Index scores by year and consumption status (*n* = 92,365)

Figure 9 shows mean Health Knowledge Index scores across all survey years by cannabis consumption. Compared to those who never consumed cannabis, scores were significantly lower among daily/almost daily consumers (β = −1.30, −1.38—−1.22; *p* < 0.001), monthly/weekly consumers (β = −1.00, −1.07—−0.92; *p* < 0.001), and those who consumed more than 1 month ago (β = −0.21, −0.25—−0.16; *p* < 0.001). Those who consumed cannabis more than 1 month ago also scored significantly higher than both monthly/weekly consumers (β = 0.79, 0.72—0.86; *p* < 0.001) and daily/almost daily consumers (β = 1.09, 1.01—1.17; *p* < 0.001). Furthermore, monthly/weekly consumers scored significantly higher than daily/almost daily consumers (β = 0.30, 0.21—0.40; *p* < 0.001).

### Noticing cannabis product health warning labels, cannabis advertising, and education campaigns

Table 3 shows the mean Health Knowledge Index score and regression coefficient from the adjusted linear regression models including each of the three policy-relevant variables: noticing health warnings, cannabis advertising, and education campaigns respectively.

**Table 3:** Effects model regressing noticing of cannabis product health warning labels, cannabis advertising, and education campaigns on Health Knowledge Index (*n* = 92,365)

Characteristic	Mean (SD)	Beta (95% CI)	*P*-value
Noticing health warning labels on products			
No	10.3 (2.7)	ref	ref
Yes	10.5 (2.5)	0.58 (0.51, 0.64)	<.001
Not applicable	10.9 (2.1)	0.26 (0.21, 0.31)	<.001
Don’t know	9.5 (2.7)	−0.47 (−0.56, −0.39)	<.001
Refuse to answer	8.4 (3.3)	−0.97 (−1.75, −0.18)	0.015
Noticing cannabis advertising			
Noticed no ads	10.7 (2.3)	ref	ref
Noticed an ad in at least 1 channel	10.4 (2.6)	−0.17 (−0.22, −0.13)	<.001
Don’t know	9.6 (2.8)	−0.75 (−0.83, −0.68)	<.001
Noticing education campaigns			
Noticed no education campaigns	10.6 (2.3)	ref	ref
Noticed an education campaign	10.5 (2.6)	0.01 (−0.03, 0.05)	0.651
Don't know	9.7 (2.7)	−0.57 (−0.64, −0.50)	<.001

Respondents who reported seeing cannabis health warnings in the past 12 months had higher Health Knowledge Index scores compared to those who reported seeing no cannabis health warnings (β = 0.58, 0.51–0.64; *p* < 0.001), those who had not seen any cannabis products or packages (β = 0.32, 0.25—0.38; *p* < 0.001), those who reported not knowing whether they saw warnings (β = 1.05, 0.96–1.15; *p* < 0.001), and those who refused to answer (β = 1.54, 0.76—2.33; *p* < 0.001). Furthermore, respondents who had not seen any cannabis products reported higher scores than those who reported not knowing whether they saw warning labels (β = 0.73, 0.65–0.82; *p* < 0.001) and those who refused to answer (β = 1.23, 0.44–2.01; *p* = 0.002).

Respondents who reported seeing cannabis advertisements in the past 12 months reported lower Health Knowledge Index scores compared to those who reported seeing no advertisements (β = −0.17, −0.22—−0.13; *p* < 0.001). In addition, respondents who reported not knowing if they had seen any cannabis advertisements reported lower scores compared to respondents who reported seeing cannabis advertisements (β = −0.58, −0.66—−0.50; *p* < 0.001) and those who reported seeing no advertisements (β = −0.75, −0.83—−0.68; *p* < 0.001).

Respondents who reported seeing an educational campaign reported higher Health Knowledge Index scores compared to those who did not know whether they had noticed any (β = 0.58, 0.51—0.66; *p* < 0.001), while scoring not significantly different compared to those who reported noticing no education campaigns (β = 0.01, −0.03 – 0.05; *p* = 0.651). In addition, those who reported noticing no education campaigns reported higher scores than those who did not know whether they had noticed any (β = 0.57, 0.50—0.64; *p* < 0.001).

Differences in Health Knowledge Index scores were also observed by cannabis consumption status and noticing of cannabis product health warning labels, cannabis advertising, and education campaigns. As shown in Table 4, scores increased with decreasing cannabis consumption frequency.

**Table 4:** Mean Health Knowledge Index scores by noticing of cannabis product health warning labels, cannabis advertising, and education campaigns and cannabis consumption status (*n* = 92,365)

Characteristic	Never consumed	Consumed more than 1 month ago	Monthly/Weekly consumption	Daily/almost daily consumption
Noticing cannabis product health warning labels				
No	10.7 (2.6)	10.6 (2.3)	9.5 (3.0)	9.0 (3.0)
Yes	11.0 (2.6)	10.6 (2.3)	10.4 (2.5)	10.1 (2.7)
Not applicable	11.1 (2.1)	10.9 (1.9)	9.2 (2.9)	8.7 (3.0)
Don’t know	9.3 (2.9)	10.0 (2.4)	9.4 (2.6)	8.9 (2.8)
Refuse to answer	8.8 (3.5)	8.6 (2.7)	7.9 (3.0)	8.4 (4.0)
Noticing cannabis advertising				
Noticed no ads	11.0 (2.2)	10.9 (2.1)	10.2 (2.3)	9.7 (2.8)
Noticed an ad in at least 1 channel	10.8 (2.4)	10.6 (2.3)	9.5 (3.0)	9.3 (3.0)
Don’t know	9.9 (2.8)	9.9 (2.5)	9.1 (2.8)	8.7 (2.9)
Noticing education campaigns				
Noticed no education campaigns	10.9 (2.2)	10.8 (2.1)	10.0 (2.5)	9.5 (2.8)
Noticed an education campaign	11.0 (2.5)	10.7 (2.3)	9.6 (3.1)	9.4 (3.0)
Don't know	9.9 (2.7)	10.0 (2.5)	9.1 (2.7)	8.9 (2.9)

## Discussion

The current study is among the first to examine changes in health knowledge of cannabis before and after cannabis legalization. Most notably, relatively few changes were observed in overall health knowledge from pre-legalization in 2018 to five years post-legalization in 2023, contrary to our hypothesis. Overall levels of health knowledge dipped in 2020 before returning to pre-legalization levels in 2023, with moderately higher levels health knowledge in 2023 after adjusting for sociodemographics and changes in cannabis use. The temporary decrease observed in 2020 may be associated with the emergence of COVID-19 pandemic, which was characterized by increases in substance use among adults, including cannabis (Lee et al., 2024; Gette et al., 2022; Boden et al., 2020). The current findings are generally consistent with trends observed in the Canadian Cannabis Survey (CCS) over the same period, in which few changes were observed in health knowledge between 2018 and 2023, except for modest increases in the percentage who agreed that cannabis was ‘habit forming’ and smoking cannabis was harmful, and decreases in agreement with the effect of frequent use on mental health (Government 2024).

Large differences were observed in the level of agreement with different health effects. Agreement was highest for the risks of impaired driving, which is a common focus for public health education campaigns in Canada. Nevertheless, one fifth of respondents did not agree that it can be dangerous to drive or operate machinery after using cannabis. Of greatest concern, only one third of respondents agreed that regular cannabis consumption can increase the risk of psychosis and schizophrenia, similar to the percentage who incorrectly reported that cannabis can cause diabetes. Consistent with the current findings, the Canadian Cannabis Survey also found the lowest levels of agreement for mental health effects, despite strong evidence to the contrary (Government 2024). Interestingly, respondents in the current study who reported previous experience of a mental health condition were more likely to agree that regular cannabis consumption can increase the risk of psychosis and schizophrenia, despite reporting lower overall scores on the health knowledge index, similar to previous findings (Thornton et al., 2013). Overall, the lack of knowledge for mental health effect warrants greater attention, particularly given higher levels of cannabis consumption among people with a history of mental health conditions compared to the general population (Rup et al., 2022; Lee et al., 2024).

As with previous studies, people that consume cannabis reported lower levels of agreement than non-consumers (Government 2024). This is consistent with a large number of studies demonstrating optimism bias among those that use substances including cannabis and tobacco (Salloum et al., 2018; Pacek et al., 2015). Other individual-level differences were observed, including greater health knowledge among younger respondents. As hypothesized, the youngest age group of 16–25-year-old respondents had the highest health knowledge compared to all other groups. Other studies also indicate that younger age groups report higher health knowledge and risk perception of cannabis compared to older ages (Han et al., 2021; Huỳnh et al., 2024). Knowledge gaps were also observed by income adequacy and education. As hypothesized, higher socioeconomic status in terms of income adequacy and educational attainment was associated with greater health knowledge. Evidence on socioeconomic status (SES) and risk perception of cannabis consumption is mixed; although one study found that SES indexed by parental education, household income, and cash-on-hand was not related to either risk assimilation or risk denial of cannabis consumption (Gette et al., 2022), others have supported an association between increased cannabis consumption and low SES (Boden et al., 2020; Cerdá, et al., 2016; Rafei et al., 2023), which may suggest a potential relationship for risk perceptions and SES. Lower levels of health knowledge among males and ‘non-white’ minority groups are also consistent with previous studies. Markedly lower levels of among respondents self-identifying as ‘black’ and ‘indigenous’—two groups that were disproportionately affected by cannabis prohibition—suggests further efforts are required to reduce cannabis-related disparities, as noted in a recent legislative review (Rosenberg 2024).

To our knowledge, the current study is the first to examine population-levels of health knowledge for cannabis and associations with risk communication policies. Noticing the mandated health warnings on cannabis products was associated with greater health knowledge. Previous studies examining the effectiveness of cannabis health warnings found that warning labels are associated with increased knowledge of health effects and increased perceived harm of smoking cannabis (Mutti-Packer et al., 2018; Pepper et al., 2020). Seeing cannabis advertisements was associated with lower health knowledge, consistent with findings from cannabis, tobacco, and alcohol research, which have indicated that positive imagery conveyed in advertising can reduce perceptions of harm (Kotnowski and Hammond 2025; Moodie and Ford 2011; Bhat et al., 2018). In contrast, noticing education campaigns was not associated with health knowledge. Few studies have examined the association between noticing public health education campaigns and cannabis risk perceptions; however, findings in Canada’s northern territories found higher risk perceptions of daily cannabis smoking associated with noticing campaigns (Schwartz et al., 2024). The effect of public education campaigns are likely to vary depending upon the prominence and effectiveness of individual media campaigns (Wakefield 2010; Hornik et al., 2008). Future research on the impact of specific public education campaigns is warranted.

### Limitations

The current study is subject to several limitations common to survey research. The study design is repeat cross-sectional and did not examine individual-level changes over time. Respondents were recruited using non-probability-based sampling; therefore, the findings do not necessarily provide nationally representative estimates. However, the data were adjusted by age group, sex, education, income adequacy, mental health status, and consumption status. Additionally, the measures of health knowledge used agreement questions and conditional causal wording such as ‘can’ when asking about health effects; which may both lead to higher estimates of health knowledge compared to open-ended, definite causal statements of health effects (Weinstein et al., 2004). Finally, the brief pre-legalization ‘baseline’ period represents a limitation. It is possible that levels of health knowledge may have been changing over a longer period, including in the build up to legalization, during which considerable media attention and public debate about the health effects of cannabis. While a longer baseline period would be an asset, the stability of health knowledge over the course of the 5-year post-legalization period covered by the current study remains notable.

## Conclusions

The current study suggests that population-level knowledge of the health knowledge of cannabis has generally remain unchanged since the legalization of recreational cannabis in 2018. The lack of change may represent a type of ‘stalemate’ between opposing forces. On the one hand, legalization has the potential to normalize cannabis consumption and undermine perceptions of risk through exposure to cannabis promotions and increased cannabis consumption. On the other hand, legalization provides greater opportunities and responsibility to convey information on the risks of cannabis consumption. This is particularly true in legal markets such as Canada, where all provincial and territorial governments serve as retailers and/or distributors for licensed cannabis products, which also provides resources for education campaigns. The lack of change in health knowledge in the first five years following legalization may allay some concerns about the immediate impact of legalization; however, it also highlights notable knowledge gaps and disparities between population subgroups that will require dedicated efforts to address. Continued monitoring is also essential given the relatively early stage of Canada’s legal market and the potential for longer term changes over time.

## Supplementary Information

Supplementary Material 1: Supplemental Table 1: Recoded health knowledge questions for Health Knowledge Index. Supplemental Table 2: Frequency of correct responses to cannabis health knowledge questions by time and cannabis consumption status (*n* = 92365). Supplemental Table 3.1: Unadjusted model examining health knowledge questions by year (*n* = 92365). Supplemental Table 3.2: Belief that marijuana smoke can be harmful (*n* = 92365). Supplemental Table 3.3: Belief that it can be harmful to use marijuana when pregnant or breastfeeding (*n* = 92365). Supplemental Table 3.4: Belief that it can be dangerous to drive or operate machinery after using marijuana (*n* = 92365). Supplemental Table 3.5: Belief that marijuana can be addictive (*n* = 92365). Supplemental Table 3.6: Belief that regular use of marijuana can increase the risk of psychosis and schizophrenia (*n* = 92365). Supplemental Table 3.7: Belief that teenagers are at greater risk of harm from using marijuana than adults (*n* = 92365). Supplemental Table 3.8: Belief that using marijuana can cause diabetes (*n* = 92365). Supplemental Table 4: Unadjusted pairwise contrasts of the Health Knowledge Index by year (*n* = 92365). Supplemental Table 5: Adjusted pairwise contrasts of the Health Knowledge Index by year (n = 92365). Supplemental Table 6: Verbatim health knowledge questions from ICPS survey. Supplemental Table 7: Mental health conditions. Supplemental Table 8: Channels of cannabis advertising and education campaigns or public health messages about marijuana.

## Data Availability

The datasets used and/or analysed during the current study are available from the corresponding author on reasonable request.
