# Supplementary material for: Knowledge about the health effects of cannabis before and after legalization of ‘recreational’ cannabis in Canada: findings from the International Cannabis Policy Study, 2018–2024
Source: J Cannabis Res. 2026 Jun 10;8:94. doi: 10.1186/s42238-026-00428-6 (PMC13483551; doi:10.1186/s42238-026-00428-6)
Supplement: Supplementary file 1 [file 42238_2026_428_MOESM1_ESM.docx]

# SUPPLEMENTAL FILES

**Supplemental Table 1: Recoded health knowledge questions for Health Knowledge Index**

| **Original ICPS question** | **Coded response** | **Response value** |
| --- | --- | --- |
| Can marijuana smoke be harmful? | No | 0 |
|  | Maybe /Don’t Know | 1 |
|  | Yes | 2 |
|  |  |  |
| Can it be harmful to use marijuana when pregnant or breastfeeding? | No | 0 |
|  | Maybe /Don’t Know | 1 |
|  | Yes | 2 |
|  |  |  |
| Can it be dangerous to drive or operate machinery after using marijuana? | No | 0 |
|  | Maybe /Don’t Know | 1 |
|  | Yes | 2 |
|  |  |  |
| Can marijuana be addictive? | No | 0 |
|  | Maybe /Don’t Know | 1 |
|  | Yes | 2 |
|  |  |  |
| Can regular use of marijuana increase the risk of psychosis and schizophrenia? | No | 0 |
|  | Maybe /Don’t Know | 1 |
|  | Yes | 2 |
|  |  |  |
| Are teenagers at greater risk of harm from using marijuana than adults? | No | 0 |
|  | Maybe /Don’t Know | 1 |
|  | Yes | 2 |
|  |  |  |
| Can using marijuana cause diabetes? | Yes | 0 |
|  | Maybe /Don’t Know | 1 |
|  | No | 2 |

**Supplemental Table 2: Frequency of correct responses to cannabis health knowledge questions by time and cannabis consumption status** (n = 92365)

| Health belief | 2018 | 2019 | 2020 | 2021 | 2022 | 2023 |
| --- | --- | --- | --- | --- | --- | --- |
|  | % (n) | % (n) | % (n) | % (n) | % (n) | % (n) |
| Can marijuana smoke be harmful? | | | | | | |
| All respondents | 58.3% (5799) | 56.6% (8492) | 55.6% (8639) | 54.4% (9079) | 55.2% (8646) | 57.8% (11292) |
| Never consumed | 67.1% (2916) | 64.8% (3722) | 60.9% (3733) | 61.7% (3914) | 62.7% (3778) | 63.5% (4747) |
| Consumed more than 1 month ago | 59.2% (2223) | 58.4% (3358) | 58.1% (3344) | 56.4% (3320) | 56.3% (3227) | 59.2% (4220) |
| Monthly/Weekly consumption | 37.6% (365) | 44.0% (818) | 44.3% (815) | 44.5% (978) | 46.0% (935) | 48.7% (1240) |
| Daily/almost daily consumption | 33.6% (295) | 35.8% (594) | 41.5% (747) | 38.4% (867) | 38.0% (706) | 45.3% (1084) |
|  |  |  |  |  |  |  |
| Can it be harmful to use marijuana when pregnant or breastfeeding? | | | | | | |
| All respondents | 73.0% (7259) | 70.8% (10630) | 69.7% (10820) | 65.1% (10860) | 67.4% (10541) | 70.2% (13717) |
| Never consumed | 77.7% (3378) | 75.5% (4335) | 72.2% (4431) | 69.7% (4422) | 72.2% (4352) | 73.0% (5457) |
| Consumed more than 1 month ago | 76.3% (2866) | 74.5% (4283) | 74.4% (4281) | 68.4% (4023) | 69.6% (3989) | 74.2% (5290) |
| Monthly/Weekly consumption | 60.2% (585) | 59.9% (1116) | 62.3% (1146) | 59.3% (1302) | 60.1% (1221) | 63.3% (1612) |
| Daily/almost daily consumption | 49.0% (431) | 54.1% (896) | 53.4% (962) | 49.2% (1113) | 52.7% (979) | 56.8% (1357) |
|  | | | | | | |
| Can it be dangerous to drive or operate machinery after using marijuana? | | | | | | |
| All respondents | 81.3% (8093) | 79.5% (11933) | 79.3% (12314) | 77.4% (12915) | 76.9% (12041) | 79.6% (15555) |
| Never consumed | 85.6% (3718) | 82.6% (4744) | 80.6% (4945) | 78.9% (5002) | 78.7% (4745) | 80.4% (6009) |
| Consumed more than 1 month ago | 85.8% (3225) | 85.1% (4891) | 85.4% (4915) | 83.7% (4926) | 81.5% (4672) | 84.9% (6053) |
| Monthly/Weekly consumption | 67.2% (653) | 71.1% (1325) | 72.4% (1333) | 71.9% (1579) | 71.3% (1448) | 72.0% (1831) |
| Daily/almost daily consumption | 56.6% (498) | 58.8% (974) | 62.3% (1121) | 62.3% (1408) | 63.2% (1176) | 69.5% (1662) |
|  | | | | | | |
| Can marijuana be addictive? | | | | | | |
| All respondents | 62.6% (6234) | 61.8% (9277) | 61.6% (9562) | 60.9% (10155) | 61.7% (9663) | 64.0% (12513) |
| Never consumed | 73.1% (3175) | 70.9% (4071) | 68.6% (4207) | 68.3% (4330) | 69.5% (4189) | 71.3% (5331) |
| Consumed more than 1 month ago | 62.8% (2359) | 63.3% (3637) | 62.8% (3618) | 63.5% (3735) | 63.3% (3630) | 65.9% (4699) |
| Monthly/Weekly consumption | 42.1% (410) | 44.8% (834) | 47.4% (873) | 46.8% (1028) | 48.9% (992) | 50.0% (1273) |
| Daily/almost daily consumption | 33.0% (290) | 44.3% (734) | 48.0% (864) | 47.0% (1062) | 45.8% (852) | 50.7% (1211) |
|  | | | | | | |
| Can regular use of marijuana increase the risk of psychosis and schizophrenia? | | | | | | |
| All respondents | 38.1% (3792) | 36.9% (5534) | 36.0% (5598) | 34.4% (5742) | 35.4% (5534) | 35.7% (6978) |
| Never consumed | 47.8% (2077) | 44.4% (2547) | 40.4% (2480) | 40.4% (2566) | 40.9% (2467) | 41.1% (3073) |
| Consumed more than 1 month ago | 35.7% (1343) | 36.6% (2103) | 36.8% (2119) | 34.1% (2007) | 34.4% (1973) | 35.6% (2534) |
| Monthly/Weekly consumption | 23.9% (232) | 28.3% (527) | 29.6% (545) | 28.0% (614) | 30.4% (617) | 28.1% (715) |
| Daily/almost daily consumption | 15.9% (140) | 21.5% (357) | 25.3% (455) | 24.6% (555) | 25.6% (476) | 27.4% (656) |
|  | | | | | | |
| Are teenagers at greater risk of harm from using marijuana than adults? | | | | | | |
| All respondents | 60.0% (5973) | 60.0% (9002) | 55.8% (8661) | 56.1% (9358) | 55.6% (8704) | 58.6% (11447) |
| Never consumed | 66.2% (2879) | 64.8% (3717) | 57.4% (3520) | 59.7% (3787) | 58.9% (3549) | 60.6% (4532) |
| Consumed more than 1 month ago | 59.7% (2245) | 61.4% (3530) | 57.3% (3297) | 56.6% (3333) | 56.6% (3244) | 60.1% (4287) |
| Monthly/Weekly consumption | 46.4% (450) | 49.6% (924) | 50.9% (936) | 51.6% (1132) | 49.8% (1012) | 52.3% (1330) |
| Daily/almost daily consumption | 45.4% (399) | 50.2% (831) | 50.5% (908) | 48.9% (1105) | 48.3% (898) | 54.3% (1298) |
|  | | | | | | |
| Can using marijuana cause diabetes? | | | | | | |
| All respondents | 36.1% (3590) | 38.4% (5765) | 37.0% (5748) | 36.9% (6148) | 34.9% (5455) | 33.4% (6524) |
| Never consumed | 24.8% (1078) | 26.2% (1507) | 23.8% (1461) | 23.7% (1503) | 23.0% (1385) | 21.4% (1597) |
| Consumed more than 1 month ago | 37.7% (1417) | 40.3% (2316) | 40.1% (2308) | 38.3% (2256) | 37.4% (2143) | 36.3% (2585) |
| Monthly/Weekly consumption | 52.0% (506) | 51.5% (960) | 50.0% (921) | 48.4% (1063) | 46.1% (936) | 42.5% (1083) |
| Daily/almost daily consumption | 67.0% (588) | 59.4% (983) | 58.8% (1058) | 58.7% (1326) | 53.3% (991) | 52.7% (1260) |

**Supplemental Table 3.1: Unadjusted model examining health knowledge questions by year** (n = 92365)

| **Characteristic** | **% Correct** | **OR (95% CI)** | **P-value** |
| --- | --- | --- | --- |
|  |  |  |  |
| **Belief that marijuana smoke can be harmful** | |  |  |
| 2018 | 58.3% | ref | ref |
| 2019 | 56.6% | 0.93 (0.87, 1.00) | 0.053 |
| 2020 | 55.6% | 0.90 (0.84, 0.96) | 0.003 |
| 2021 | 54.4% | 0.86 (0.80, 0.92) | <.001 |
| 2022 | 55.2% | 0.88 (0.83, 0.95) | <.001 |
| 2023 | 57.8% | 0.98 (0.92, 1.05) | 0.570 |
|  |  |  |  |
| **Belief that it can be harmful to use marijuana when pregnant or breastfeeding** | |  |  |
| 2018 | 72.9% | ref | ref |
| 2019 | 70.8% | 0.90 (0.84, 0.97) | 0.008 |
| 2020 | 69.7% | 0.85 (0.79, 0.92) | <.001 |
| 2021 | 65.1% | 0.69 (0.64, 0.75) | <.001 |
| 2022 | 67.4% | 0.77 (0.71, 0.83) | <.001 |
| 2023 | 70.2% | 0.87 (0.81, 0.94) | <.001 |
|  |  |  |  |
| **Belief that it can be dangerous to drive or operate machinery after using marijuana** | |  |  |
| 2018 | 81.3% | ref | ref |
| 2019 | 79.5% | 0.89 (0.82, 0.98) | 0.013 |
| 2020 | 79.3% | 0.88 (0.80, 0.96) | 0.006 |
| 2021 | 77.4% | 0.79 (0.72, 0.86) | <.001 |
| 2022 | 76.9% | 0.77 (0.70, 0.84) | <.001 |
| 2023 | 79.6% | 0.90 (0.82, 0.98) | 0.015 |
|  |  |  |  |
| **Belief that marijuana can be addictive** | |  |  |
| 2018 | 62.6% | ref | ref |
| 2019 | 61.8% | 0.97 (0.90, 1.04) | 0.333 |
| 2020 | 61.6% | 0.96 (0.89, 1.03) | 0.211 |
| 2021 | 60.9% | 0.93 (0.87, 0.99) | 0.033 |
| 2022 | 61.7% | 0.96 (0.90, 1.03) | 0.288 |
| 2023 | 64.0% | 1.06 (0.99, 1.14) | 0.081 |
|  |  |  |  |
| **Belief that regular use of marijuana can increase the risk of psychosis and schizophrenia** | |  |  |
| 2018 | 38.1% | ref | ref |
| 2019 | 36.9% | 0.95 (0.89, 1.02) | 0.144 |
| 2020 | 36.0% | 0.92 (0.85, 0.98) | 0.015 |
| 2021 | 34.4% | 0.85 (0.80, 0.91) | <.001 |
| 2022 | 35.4% | 0.89 (0.83, 0.95) | 0.001 |
| 2023 | 35.7% | 0.90 (0.84, 0.97) | 0.003 |
|  |  |  |  |
| **Belief that teenagers are at greater risk of harm from using marijuana than adults** | |  |  |
| 2018 | 60.0% | ref | ref |
| 2019 | 60.0% | 1.00 (0.93, 1.07) | 0.982 |
| 2020 | 55.8% | 0.84 (0.78, 0.90) | <.001 |
| 2021 | 56.1% | 0.85 (0.80, 0.91) | <.001 |
| 2022 | 55.6% | 0.84 (0.78, 0.90) | <.001 |
| 2023 | 58.6% | 0.94 (0.88, 1.01) | 0.087 |
|  |  |  |  |
| **Belief that using marijuana can cause diabetes** | |  |  |
| 2018 | 36.1% | ref | ref |
| 2019 | 38.4% | 1.11 (1.03, 1.19) | 0.005 |
| 2020 | 37.0% | 1.04 (0.97, 1.12) | 0.258 |
| 2021 | 36.9% | 1.04 (0.97, 1.11) | 0.333 |
| 2022 | 34.9% | 0.95 (0.89, 1.02) | 0.139 |
| 2023 | 33.4% | 0.89 (0.83, 0.95) | 0.001 |

**Supplemental Table 3.2:** **Belief that marijuana smoke can be harmful** (n = 92365)

| Characteristic | % Correct | AOR (95% CI) | P-value |
| --- | --- | --- | --- |
|  |  |  |  |
| Survey year |  |  |  |
| 2018 | 58.3% | ref | ref |
| 2019 | 56.6% | 1.01 (0.94, 1.09) | 0.727 |
| 2020 | 55.6% | 0.98 (0.91, 1.05) | 0.551 |
| 2021 | 54.4% | 0.96 (0.90, 1.03) | 0.286 |
| 2022 | 55.2% | 1.02 (0.95, 1.09) | 0.578 |
| 2023 | 57.8% | 0.98 (0.91, 1.05) | 0.012 |
|  |  |  |  |
| Cannabis Consumption |  |  |  |
| Never consumed | 63.2% | ref | ref |
| Consumed more than 1 month ago | 57.9% | 0.77 (0.74, 0.80) | <.001 |
| Monthly/Weekly consumption | 45.0% | 0.48 (0.45, 0.51) | <.001 |
| Daily/almost daily consumption | 39.6% | 0.41 (0.38, 0.43) | <.001 |
|  |  |  |  |
| Mental Health Diagnosis |  |  |  |
| No | 59.3% | ref | ref |
| Yes | 53.3% | -0.08 (-0.12, -0.04) | <.001 |
| Not stated* | 38.8% | -0.63 (-0.71, -0.54) | <.001 |
|  |  |  |  |
| Age |  |  |  |
| 16-25 years | 57.1% | ref | ref |
| 26-35 years | 50.1% | 0.72 (0.68, 0.77) | <.001 |
| 36-45 years | 54.7% | 0.81 (0.76, 0.86) | <.001 |
| 46-55 years | 59.4% | 0.91 (0.86, 0.97) | 0.003 |
| 56-65 years | 60.2% | 0.91 (0.86, 0.97) | 0.003 |
|  |  |  |  |
| Sex |  |  |  |
| Female | 59.4% | ref | ref |
| Male | 57.8% | 0.95 (0.92, 0.99) | 0.007 |
|  |  |  |  |
| Ethnicity |  |  |  |
| White only | 60.2% | ref | ref |
| East/South East Asian only | 60.5% | 0.88 (0.83, 0.93) | <.001 |
| Mixed/Other/Unstated | 52.4% | 0.82 (0.77, 0.89) | <.001 |
| South Asian only | 56.2% | 0.77 (0.71, 0.84) | <.001 |
| Black only | 53.1% | 0.73 (0.66, 0.80) | <.001 |
| Indigenous only | 46.1% | 0.71 (0.63, 0.79) | <.001 |
| Latino only | 48.0% | 0.60 (0.53, 0.69) | <.001 |
| Middle Eastern only | 58.9% | 0.94 (0.83, 1.07) | 0.360 |
|  |  |  |  |
| Income Adequacy |  |  |  |
| Very difficult | 53.3% | ref | ref |
| Difficult | 55.6% | 1.03 (0.96, 1.10) | 0.484 |
| Neither easy nor difficult | 54.3% | 0.91 (0.86, 0.97) | 0.006 |
| Easy | 60.6% | 1.12 (1.05, 1.20) | 0.001 |
| Very easy | 62.6% | 1.20 (1.11, 1.30) | <.001 |
| Not stated* | 43.2% | 0.66 (0.59, 0.75) | <.001 |
|  |  |  |  |
| Education |  |  |  |
| Less than high school | 53.1% | ref |  |
| High school diploma or equivalent | 50.3% | -0.06 (-0.13, 0.01) | 0.109 |
| Bachelor's degree or higher | 60.1% | 0.33 (0.27, 0.39) | <.001 |
| Not stated* | 36.2% | -0.32 (-0.51, -0.14) | 0.001 |

**Supplemental Table 3.3: Belief that it can be harmful to use marijuana when pregnant or breastfeeding** (n = 92365)

| **Characteristic** | **% Correct** | **AOR (95% CI)** | **P-value** |
| --- | --- | --- | --- |
|  |  |  |  |
| **Survey year** |  |  |  |
| 2018 | 72.9% | ref | ref |
| 2019 | 70.8% | 0.99 (0.91, 1.07) | 0.757 |
| 2020 | 69.7% | 0.95 (0.88, 1.03) | 0.186 |
| 2021 | 65.1% | 0.79 (0.73, 0.85) | <.001 |
| 2022 | 67.4% | 0.87 (0.81, 0.94) | <.001 |
| 2023 | 70.2% | 1.00 (0.92, 1.08) | 0.944 |
|  |  |  |  |
| **Cannabis Consumption** |  |  |  |
| Never consumed | 73.1% | ref | ref |
| Consumed more than 1 month ago | 72.7% | 0.93 (0.89, 0.98) | 0.003 |
| Monthly/Weekly consumption | 61.0% | 0.61 (0.58, 0.65) | <.001 |
| Daily/almost daily consumption | 52.9% | 0.45 (0.42, 0.48) | <.001 |
|  |  |  |  |
| **Mental Health Diagnosis** |  |  |  |
| No | 72.2% | ref | ref |
| Yes | 66.7% | -0.17 (-0.22, -0.13) | <.001 |
| Not stated* | 47.2% | -0.82 (-0.91, -0.73) | <.001 |
|  |  |  |  |
| **Age** |  |  |  |
| 16-25 years | 70.4% | ref | ref |
| 26-35 years | 61.8% | 0.66 (0.62, 0.71) | <.001 |
| 36-45 years | 66.2% | 0.75 (0.70, 0.80) | <.001 |
| 46-55 years | 73.4% | 0.97 (0.90, 1.04) | 0.329 |
| 56-65 years | 74.0% | 0.94 (0.88, 1.00) | 0.047 |
|  |  |  |  |
| **Sex** |  |  |  |
| Female | 74.8% | ref | ref |
| Male | 65.8% | 0.67 (0.64, 0.69) | <.001 |
|  |  |  |  |
| **Ethnicity** |  |  |  |
| White only | 73.1% | ref | ref |
| East/South East Asian only | 70.0% | 0.79 (0.74, 0.84) | <.001 |
| Mixed/Other/Unstated | 63.5% | 0.78 (0.73, 0.85) | <.001 |
| South Asian only | 66.6% | 0.71 (0.65, 0.78) | <.001 |
| Black only | 65.1% | 0.69 (0.62, 0.76) | <.001 |
| Indigenous only | 61.8% | 0.76 (0.68, 0.85) | <.001 |
| Latino only | 68.4% | 0.81 (0.70, 0.94) | 0.005 |
| Middle Eastern only | 67.2% | 0.81 (0.71, 0.93) | 0.003 |
|  |  |  |  |
| **Income Adequacy** |  |  |  |
| Very difficult | 67.3% | ref | ref |
| Difficult | 69.4% | 1.05 (0.97, 1.13) | 0.238 |
| Neither easy nor difficult | 68.6% | 0.97 (0.90, 1.04) | 0.386 |
| Easy | 71.7% | 1.09 (1.01, 1.17) | 0.032 |
| Very easy | 72.1% | 1.11 (1.02, 1.21) | 0.019 |
| Not stated* | 52.9% | 0.58 (0.52, 0.66) | <.001 |
|  |  |  |  |
| **Education** |  |  |  |
| Less than high school | 67.7% | ref | ref |
| High school diploma or equivalent | 67.2% | -0.02 (-0.10, 0.05) | 0.594 |
| Bachelor's degree or higher | 70.7% | 0.14 (0.07, 0.20) | <.001 |
| Not stated* | 44.0% | -0.46 (-0.65, -0.28) | <.001 |

**Supplemental Table 3.4: Belief that it can be dangerous to drive or operate machinery after using marijuana** (n = 92365)

| **Characteristic** | **% Correct** | **AOR (95% CI)** | **P-value** |
| --- | --- | --- | --- |
|  |  |  |  |
| **Survey year** |  |  |  |
| 2018 | 81.3% | ref | ref |
| 2019 | 79.5% | 1.01 (0.92, 1.10) | 0.919 |
| 2020 | 79.3% | 1.03 (0.94, 1.13) | 0.566 |
| 2021 | 77.4% | 0.97 (0.88, 1.06) | 0.473 |
| 2022 | 76.9% | 0.92 (0.84, 1.01) | 0.076 |
| 2023 | 79.6% | 1.09 (1.00, 1.20) | 0.053 |
|  |  |  |  |
| **Cannabis Consumption** |  |  |  |
| Never consumed | 80.9% | ref | ref |
| Consumed more than 1 month ago | 84.3% | 1.13 (1.07, 1.19) | <.001 |
| Monthly/Weekly consumption | 71.4% | 0.60 (0.56, 0.64) | <.001 |
| Daily/almost daily consumption | 63.1% | 0.41 (0.38, 0.44) | <.001 |
|  |  |  |  |
| **Mental Health Diagnosis** |  |  |  |
| No | 81.6% | ref | ref |
| Yes | 77.7% | -0.13 (-0.18, -0.08) | <.001 |
| Not stated* | 53.4% | -1.03 (-1.12, -0.93) | <.001 |
|  |  |  |  |
| **Age** |  |  |  |
| 16-25 years | 78.5% | ref | ref |
| 26-35 years | 70.6% | 0.58 (0.54, 0.63) | <.001 |
| 36-45 years | 76.0% | 0.69 (0.64, 0.75) | <.001 |
| 46-55 years | 83.2% | 0.97 (0.89, 1.05) | 0.431 |
| 56-65 years | 86.1% | 1.09 (1.00, 1.17) | 0.044 |
|  |  |  |  |
| **Sex** |  |  |  |
| Female | 84.2% | ref | ref |
| Male | 78.1% | 0.70 (0.67, 0.73) | <.001 |
|  |  |  |  |
| **Ethnicity** |  |  |  |
| White only | 84.8% | ref | ref |
| East/South East Asian only | 78.7% | 0.67 (0.62, 0.72) | <.001 |
| Mixed/Other/Unstated | 73.2% | 0.66 (0.61, 0.72) | <.001 |
| South Asian only | 69.2% | 0.44 (0.40, 0.49) | <.001 |
| Black only | 70.4% | 0.48 (0.43, 0.53) | <.001 |
| Indigenous only | 72.1% | 0.63 (0.56, 0.72) | <.001 |
| Latino only | 75.7% | 0.63 (0.54, 0.73) | <.001 |
| Middle Eastern only | 70.8% | 0.49 (0.42, 0.57) | <.001 |
|  |  |  |  |
| **Income Adequacy** |  |  |  |
| Very difficult | 76.8% | ref | ref |
| Difficult | 79.5% | 1.11 (1.01, 1.21) | 0.022 |
| Neither easy nor difficult | 78.2% | 0.98 (0.90, 1.06) | 0.619 |
| Easy | 82.0% | 1.17 (1.07, 1.28) | 0.001 |
| Very easy | 81.7% | 1.11 (1.01, 1.23) | 0.041 |
| Not stated* | 59.6% | 0.54 (0.48, 0.62) | <.001 |
|  |  |  |  |
| **Education** |  |  |  |
| Less than high school | 76.3% | ref | ref |
| High school diploma or equivalent | 76.8% | 0.00 (-0.08, 0.09) | 0.947 |
| Bachelor's degree or higher | 81.0% | 0.28 (0.20, 0.36) | <.001 |
| Not stated* | 45.9% | -0.65 (-0.85, -0.45) | <.001 |

**Supplemental Table 3.5: Belief that marijuana can be addictive** (n = 92365)

| **Characteristic** | **% Correct** | **AOR (95% CI)** | **P-value** |
| --- | --- | --- | --- |
|  |  |  |  |
| **Survey year** |  |  |  |
| 2018 | 62.6% | ref | ref |
| 2019 | 61.8% | 1.05 (0.98, 1.13) | 0.182 |
| 2020 | 61.6% | 1.05 (0.98, 1.13) | 0.151 |
| 2021 | 60.9% | 1.06 (0.99, 1.13) | 0.126 |
| 2022 | 61.7% | 1.08 (1.00, 1.16) | 0.044 |
| 2023 | 64.0% | 1.18 (1.10, 1.23) | <.001 |
|  |  |  |  |
| **Cannabis Consumption** |  |  |  |
| Never consumed | 70.2% | ref | ref |
| Consumed more than 1 month ago | 63.7% | 0.75 (0.72, 0.79) | <.001 |
| Monthly/Weekly consumption | 47.3% | 0.40 (0.37, 0.42) | <.001 |
| Daily/almost daily consumption | 46.2% | 0.38 (0.36,0.41) | <.001 |
|  |  |  |  |
| **Mental Health Diagnosis** |  |  |  |
| No | 65.0% | ref | ref |
| Yes | 59.7% | -0.13 (-0.17, -0.09) | <.001 |
| Not stated* | 44.7% | -0.71 (-0.80, -0.62) | <.001 |
|  |  |  |  |
| **Age** |  |  |  |
| 16-25 years | 67.3% | ref | ref |
| 26-35 years | 57.0% | 0.70 (0.65, 0.74) | <.001 |
| 36-45 years | 59.8% | 0.73 (0.68, 0.78) | <.001 |
| 46-55 years | 65.5% | 0.87 (0.81, 0.93) | <.001 |
| 56-65 years | 61.9% | 0.71 (0.67, 0.76) | <.001 |
|  |  |  |  |
| **Sex** |  |  |  |
| Female | 65.9% | ref | ref |
| Male | 57.9% | 0.74 (0.72, 0.77) | <.001 |
|  |  |  |  |
| **Ethnicity** |  |  |  |
| White only | 62.4% | ref | ref |
| East/South East Asian only | 67.5% | 1.02 (0.95, 1.08) | 0.645 |
| Mixed/Other/Unstated | 57.4% | 0.86 (0.80, 0.93) | <.001 |
| South Asian only | 65.6% | 0.94 (0.86, 1.02) | 0.147 |
| Black only | 68.6% | 1.09 (0.99, 1.21) | 0.075 |
| Indigenous only | 56.9% | 0.94 (0.84, 1.05) | 0.282 |
| Latino only | 64.4% | 0.98 (0.85, 1.13) | 0.774 |
| Middle Eastern only | 62.1% | 0.89 (0.78, 1.02) | 0.104 |
|  |  |  |  |
| **Income Adequacy** |  |  |  |
| Very difficult | 63.3% | Ref | ref |
| Difficult | 63.4% | 0.95 (0.89, 1.02) | 0.189 |
| Neither easy nor difficult | 61.3% | 0.83 (0.77, 0.88) | <.001 |
| Easy | 63.4% | 0.89 (0.83, 0.95) | 0.001 |
| Very easy | 62.6% | 0.86 (0.79, 0.93) | <.001 |
| Not stated* | 50.7% | 0.54 (0.48, 0.61) | <.001 |
|  |  |  |  |
| **Education** |  |  |  |
| Less than high school | 64.0% | ref | ref |
| High school diploma or equivalent | 60.7% | -0.06 (-0.13, 0.01) | 0.091 |
| Bachelor's degree or higher | 62.7% | 0.00 (-0.07, 0.06) | 0.985 |
| Not stated* | 42.2% | -0.52 (-0.70, -0.33) | <.001 |

**Supplemental Table 3.6: Belief that regular use of marijuana can increase the risk of psychosis and schizophrenia** (n = 92365)

| **Characteristic** | **% Correct** | **AOR (95% CI)** | **P-value** |
| --- | --- | --- | --- |
|  |  |  |  |
| **Survey year** |  |  |  |
| 2018 | 38.1% | ref | ref |
| 2019 | 36.9% | 1.00 (0.93, 1.07) | 0.918 |
| 2020 | 36.0% | 0.97 (0.90, 1.04) | 0.332 |
| 2021 | 34.4% | 0.91 (0.85, 0.98) | 0.010 |
| 2022 | 35.4% | 0.94 (0.87, 1.01) | 0.073 |
| 2023 | 35.7% | 0.95 (0.89, 1.02) | 0.158 |
|  |  |  |  |
| **Cannabis Consumption** |  |  |  |
| Never consumed | 42.2% | ref | ref |
| Consumed more than 1 month ago | 35.5% | 0.73 (0.70, 0.76) | <.001 |
| Monthly/Weekly consumption | 28.4% | 0.52 (0.49, 0.55) | <.001 |
| Daily/almost daily consumption | 24.3% | 0.43 (0.40, 0.46) | <.001 |
|  |  |  |  |
| **Mental Health Diagnosis** |  |  |  |
| No | 36.8% | ref | ref |
| Yes | 35.9% | 0.08 (0.04, 0.12) | <.001 |
| Not stated* | 25.0% | -0.45 (-0.54, -0.35) | <.001 |
|  |  |  |  |
| **Age** |  |  |  |
| 16-25 years | 39.2% | ref | ref |
| 26-35 years | 34.7% | 0.84 (0.78, 0.89) | <.001 |
| 36-45 years | 35.6% | 0.83 (0.78, 0.88) | <.001 |
| 46-55 years | 36.9% | 0.83 (0.78, 0.88) | <.001 |
| 56-65 years | 33.7% | 0.71 (0.67, 0.76) | <.001 |
|  |  |  |  |
| **Sex** |  |  |  |
| Female | 36.8% | ref | ref |
| Male | 34.6% | 0.96 (0.93, 1.00) | 0.033 |
|  |  |  |  |
| **Ethnicity** |  |  |  |
| White only | 35.9% | ref | ref |
| East/South East Asian only | 37.4% | 0.83 (0.78, 0.88) | <.001 |
| Mixed/Other/Unstated | 34.3% | 0.93 (0.87, 1.01) | 0.071 |
| South Asian only | 38.0% | 0.86 (0.79, 0.94) | 0.001 |
| Black only | 38.5% | 0.93 (0.85, 1.03) | 0.155 |
| Indigenous only | 29.5% | 0.89 (0.79, 1.00) | 0.048 |
| Latino only | 33.9% | 0.79 (0.69, 0.91) | 0.001 |
| Middle Eastern only | 40.5% | 1.07 (0.94, 1.22) | 0.298 |
|  |  |  |  |
| **Income Adequacy** |  |  |  |
| Very difficult | 36.7% | ref | ref |
| Difficult | 34.9% | 0.89 (0.83, 0.95) | 0.001 |
| Neither easy nor difficult | 34.3% | 0.84 (0.79 ,0.90) | <.001 |
| Easy | 38.4% | 0.96 (0.90, 1.03) | 0.306 |
| Very easy | 40.6% | 1.06 (0.98, 1.15) | 0.132 |
| Not stated* | 27.4% | 0.60 (0.53, 0.68) | <.001 |
|  |  |  |  |
| **Education** |  |  |  |
| Less than high school | 35.6% | ref | ref |
| High school diploma or equivalent | 31.6% | -0.07 (-0.15, 0.00) | 0.043 |
| Bachelor's degree or higher | 38.1% | 0.20 (0.13, 0.26) | <.001 |
| Not stated* | 26.2% | -0.17 (-0.37, 0.03) | 0.104 |

**Supplemental Table 3.7: Belief that teenagers are at greater risk of harm from using marijuana than adults** (n = 92365)

| **Characteristic** | **% Correct** | **AOR (95% CI)** | **P-value** |
| --- | --- | --- | --- |
|  |  |  |  |
| **Survey year** |  |  |  |
| 2018 | 60.0% | ref | ref |
| 2019 | 60.0% | 1.04 (0.97, 1.12) | 0.229 |
| 2020 | 55.8% | 0.88 (0.82, 0.94) | <.001 |
| 2021 | 56.1% | 0.90 (0.84, 0.97) | 0.003 |
| 2022 | 55.6% | 0.87 (0.81, 0.93) | <.001 |
| 2023 | 58.6% | 0.98 (0.92, 1.05) | 0.633 |
|  |  |  |  |
| **Cannabis Consumption** |  |  |  |
| Never consumed | 61.0% | ref | ref |
| Consumed more than 1 month ago | 58.6% | 0.85 (0.82, 0.89) | <.001 |
| Monthly/Weekly consumption | 50.5% | 0.64 (0.61, 0.68) | <.001 |
| Daily/almost daily consumption | 50.2% | 0.67 (0.63, 0.71) | <.001 |
|  |  |  |  |
| **Mental Health Diagnosis** |  |  |  |
| No | 58.6% | ref | ref |
| Yes | 58.0% | 0.07 (0.03, 0.11) | 0.001 |
| Not stated* | 40.1% | -0.55 (-0.63, -0.46) | <.001 |
|  |  |  |  |
| **Age** |  |  |  |
| 16-25 years | 57.3% | ref | ref |
| 26-35 years | 53.8% | 0.75 (0.70, 0.80) | <.001 |
| 36-45 years | 56.9% | 0.82 (0.77, 0.87) | <.001 |
| 46-55 years | 60.2% | 0.91 (0.85, 0.96) | 0.002 |
| 56-65 years | 59.6% | 0.88 (0.83, 0.94) | <.001 |
|  |  |  |  |
| **Sex** |  |  |  |
| Female | 62.5% | ref | ref |
| Male | 57.8% | 0.86 (0.83, 0.89) | <.001 |
|  |  |  |  |
| **Ethnicity** |  |  |  |
| White only | 61.8% | ref | ref |
| East/South East Asian only | 60.8% | 0.90 (0.85, 0.96) | <.001 |
| Mixed/Other/Unstated | 54.7% | 0.89 (0.83, 0.96) | 0.001 |
| South Asian only | 59.4% | 0.93 (0.85, 1.02) | 0.104 |
| Black only | 56.3% | 0.85 (0.77, 0.93) | <.001 |
| Indigenous only | 53.4% | 0.86 (0.77, 0.96) | 0.005 |
| Latino only | 55.7% | 0.85 (0.75, 0.97) | 0.017 |
| Middle Eastern only | 59.2% | 1.01 (0.88, 1.15) | 0.894 |
|  |  |  |  |
| **Income Adequacy** |  |  |  |
| Very difficult | 56.7% | ref | ref |
| Difficult | 57.5% | 0.99 (0.93, 1.06) | 0.835 |
| Neither easy nor difficult | 56.0% | 0.92 (0.86, 0.98) | 0.011 |
| Easy | 61.2% | 1.10 (1.03, 1.18) | 0.006 |
| Very easy | 61.8% | 1.12 (1.04, 1.21) | 0.004 |
| Not stated* | 39.8% |  | <.001 |
|  |  |  |  |
| **Education** |  |  |  |
| Less than high school | 51.7% | ref | ref |
| High school diploma or equivalent | 52.4% | 0.07 (0.00, 0.14) | 0.044 |
| Bachelor's degree or higher | 61.6% | 0.45 (0.39, 0.51) | <.001 |
| Not stated* | 38.1% | -0.07 (-0.26, 0.11) | 0.428 |

**Supplemental Table 3.8: Belief that using marijuana can cause diabetes** (n = 92365)

| **Characteristic** | **% Correct** | **AOR (95% CI)** | **P-value** |
| --- | --- | --- | --- |
|  |  |  |  |
| **Survey year** |  |  |  |
| 2018 | 36.1% | ref | ref |
| 2019 | 38.4% | 1.06 (0.99, 1.14) | 0.095 |
| 2020 | 37.0% | 1.02 (0.95, 1.10) | 0.642 |
| 2021 | 36.9% | 0.98 (0.91, 1.05) | 0.608 |
| 2022 | 34.9% | 0.91 (0.85, 0.98) | 0.016 |
| 2023 | 33.4% | 0.85 (0.79, 0.91) | <.001 |
|  |  |  |  |
| **Cannabis Consumption** |  |  |  |
| Never consumed | 23.7% | ref | ref |
| Consumed more than 1 month ago | 38.3% | 1.79 (1.71, 1.87) | <.001 |
| Monthly/Weekly consumption | 47.8% | 2.71 (2.55, 2.87) | <.001 |
| Daily/almost daily consumption | 57.2% | 3.81 (3.57, 4.06) | <.001 |
|  |  |  |  |
| **Mental Health Diagnosis** |  |  |  |
| No | 33.2% | ref | ref |
| Yes | 42.1% | 0.11 (0.07, 0.15) | <.001 |
| Not stated* | 25.9% | -0.32 (-0.42, -0.22) | <.001 |
|  |  |  |  |
| **Age** |  |  |  |
| 16-25 years | 31.8% | ref | ref |
| 26-35 years | 39.3% | 1.08 (1.01, 1.16) | 0.021 |
| 36-45 years | 37.9% | 1.05 (0.98, 1.12) | 0.150 |
| 46-55 years | 35.0% | 0.95 (0.89, 1.02) | 0.140 |
| 56-65 years | 35.3% | 0.96 (0.90, 1.02) | 0.153 |
|  |  |  |  |
| **Sex** |  |  |  |
| Female | 37.8% | ref | ref |
| Male | 35.0% | 0.89 (0.86, 0.93) | <.001 |
|  |  |  |  |
| **Ethnicity** |  |  |  |
| White only | 39.6% | ref | ref |
| East/South East Asian only | 24.2% | 0.63 (0.59, 0.68) | <.001 |
| Mixed/Other/Unstated | 33.8% | 0.83 (0.77, 0.90) | <.001 |
| South Asian only | 20.8% | 0.53 (0.48, 0.59) | <.001 |
| Black only | 28.2% | 0.67 (0.61, 0.75) | <.001 |
| Indigenous only | 47.6% | 1.11 (0.99, 1.24) | 0.078 |
| Latino only | 36.1% | 0.89 (0.78, 1.02) | 0.097 |
| Middle Eastern only | 23.4% | 0.58 (0.50, 0.67) | <.001 |
|  |  |  |  |
| **Income Adequacy** |  |  |  |
| Very difficult | 38.4% | ref | ref |
| Difficult | 38.8% | 1.07 (1.00, 1.15) | 0.054 |
| Neither easy nor difficult | 34.9% | 1.00 (0.93, 1.07) | 0.937 |
| Easy | 36.3% | 1.08 (1.00, 1.16) | 0.040 |
| Very easy | 35.9% | 1.06 (0.98, 1.15) | 0.149 |
| Not stated* | 20.2% | 0.59 (0.52, 0.68) | <.001 |
|  |  |  |  |
| **Education** |  |  |  |
| Less than high school | 35.8% | ref | ref |
| High school diploma or equivalent | 36.5% | -0.08 (-0.15, 0.00) | 0.039 |
| Bachelor's degree or higher | 36.1% | 0.00 (-0.06, 0.06) | 0.001 |
| Not stated* | 18.4% | -0.38 (-0.60, -0.15) | 0.001 |

**Supplemental Table 4:** **Unadjusted pairwise contrasts of the Health Knowledge Index by year** (n = 92365)

| **Reference** | **Comparison** | **Beta (95% CI)** | **P-value** |
| --- | --- | --- | --- |
| **2018** | 2019 | -0.04 (-0.12, 0.05) | 0.375 |
|  | 2020 | -0.12 (-0.21, -0.03) | 0.006 |
|  | 2021 | -0.25 (-0.33, -0.17) | <.001 |
|  | 2022 | -0.23 (-0.32, -0.17) | <.001 |
|  | 2023 | -0.04 (-0.12, 0.04) | 0.329 |
| **2019** | 2020 | 0.08 (0.01, 0.16) | 0.029 |
|  | 2021 | 0.21 (0.14, 0.28) | <.001 |
|  | 2022 | 0.19 (0.12, 0.26) | <.001 |
|  | 2023 | 0.00 (-0.07, 0.07) | 0.950 |
| **2020** | 2021 | 0.13 (0.06, 0.20) | 0.001 |
|  | 2022 | 0.11 (0.04, 0.18) | 0.003 |
|  | 2023 | -0.08 (-0.15, -0.01) | 0.025 |
| **2021** | 2022 | -0.02 (-0.09, 0.05) | 0.606 |
|  | 2023 | -0.21 (-0.28, -0.14) | <.001 |
| **2022** | 2023 | -0.19 (-0.26, -0.12) | <.001 |

**Supplemental Table 5:** **Adjusted pairwise contrasts of the Health Knowledge Index by year** (n = 92365)

| **Reference** | **Comparison** | **Beta (95% CI)** | **P-value** |
| --- | --- | --- | --- |
| **2018** | 2019 | 0.09 (0.01, 0.17) | 0.032 |
|  | 2020 | 0.03 (-0.05, 0.11) | 0.478 |
|  | 2021 | -0.04 (-0.12, 0.04) | 0.319 |
|  | 2022 | -0.05 (-0.13, 0.03) | 0.255 |
|  | 2023 | 0.14 (0.06, 0.22) | <.001 |
| **2019** | 2020 | 0.06 (-0.01, 0.13) | 0.100 |
|  | 2021 | 0.13 (0.06, 0.20) | <.001 |
|  | 2022 | 0.14 (0.07, 0.20) | <.001 |
|  | 2023 | -0.05 (-0.12, 0.01) | 0.121 |
| **2020** | 2021 | 0.07 (0.00, 0.14) | 0.046 |
|  | 2022 | 0.08 (0.01, 0.15) | 0.031 |
|  | 2023 | -0.11 (-0.18, -0.04) | 0.001 |
| **2021** | 2022 | 0.01 (-0.06, 0.07) | 0.863 |
|  | 2023 | -0.18 (-0.25, -0.12) | <.001 |
| **2022** | 2023 | -0.19 (-0.25, -0.12) | <.001 |

**Supplemental Table 6: Verbatim health knowledge questions from ICPS survey**

**Health knowledge question**

Can marijuana smoke be harmful?

Can it be harmful to use marijuana when pregnant or breastfeeding?

Can it be dangerous to drive or operate machinery after using marijuana?

Can marijuana be addictive?

Can regular use of marijuana increase the risk of psychosis and schizophrenia

Are teenagers at greater risk of harm from using marijuana than adults?

*(Bogus question)* Can using marijuana cause diabetes?

**Supplemental Table 7: Mental health conditions**

**Mental health condition**

Anxiety (including phobia, obsessive-compulsive disorder or a panic disorder

Depression (including dysthymia)

Post-traumatic stress disorder (PTSD) or traumatic event (e.g., abuse or loss)

Bipolar disorder, mania, or borderline personality disorder

Psychosis (e.g., paranoia, disorganized thinking, hearing voices that others can’t hear) or Dissociative Identity Disorder

Schizophrenia

Alcohol or other drug use

Eating disorder

ADD/ADHD

Other significant emotional or mental health problem

Other: Stress

**Supplemental Table 8: Channels of cannabis advertising and education campaigns or public health messages about marijuana**

**Cannabis advertising**

Regular postal mail

Websites or Other: Online (incl. news and smartphone ads

Email or text messages

Bars, pubs, or nightclubs

Inside stores that sell marijuana or Other: inside other establishments selling marijuana (gas stations, grocery or convenience stores, etc.)

Outside stores that sell marijuana

Outside stores that sell marijuana

Events like sporting events, concerts, festivals or markets

Kiosks or temporary sales locations (in shopping centers or on the street)

TV or radio

Billboards or posters or Other: Highway/road signs

Print newspapers or magazines

At the movies

Taxis or buses/public transit or Other: Other vehicles

Flyers

Social media, like Facebook, Twitter, YouTube, Instagram or Snapchat or Other: apps

Other

**Education campaigns or public health messages about marijuana**

School

Work

Websites

Email or text messages

Bars, pubs or nightclubs

Inside stores that sell marijuana

Outside stores that sell marijuana

Pharmacies

Events like sporting events, concerts, festivals or markets

Kiosks or temporary sales locations (in shopping centers or on the street)

TV or radio

Billboards or posters

Print newspapers or magazines

At the movies

Taxis or buses/public transit

Flyers

Social media, like Facebook, Twitter, YouTube, Instagram or Snapchat

Other (please specify):
